# Constitutively activated AMPKα1 protects against skeletal aging in mice by promoting bone‐derived IGF‐1 secretion

**DOI:** 10.1111/cpr.13476

**Published:** 2023-04-11

**Authors:** Yiqi Yang, Kai Yuan, Yihao Liu, Qishan Wang, Yixuan Lin, Shengbing Yang, Kai Huang, Tianyou Kan, Yuxin Zhang, Mingming Xu, Zhifeng Yu, Qiming Fan, Yugang Wang, Hanjun Li, Tingting Tang

**Affiliations:** ^1^ Shanghai Key Laboratory of Orthopaedic Implants, Department of Orthopaedic Surgery, Shanghai Ninth People's Hospital Shanghai Jiao Tong University School of Medicine Shanghai China; ^2^ School of Pharmacy Shanghai Jiao Tong University Shanghai China; ^3^ Department of Rehabilitation Medicine, Shanghai Ninth People's Hospital Shanghai Jiao Tong University School of Medicine Shanghai China; ^4^ Department of Trauma Surgery, Department of Orthopedics, Renji Hospital, School of Medicine Shanghai Jiao Tong University Shanghai China; ^5^ Clinical Stem Cell Research Center, Renji Hospital Shanghai Jiao Tong University School of Medicine Shanghai China

## Abstract

Senile osteoporosis is characterized by age‐related bone loss and bone microarchitecture deterioration. However, little is known to date about the mechanism that maintains bone homeostasis during aging. In this study, we identify adenosine monophosphate‐activated protein kinase alpha 1 (AMPKα1) as a critical factor regulating the senescence and lineage commitment of mesenchymal stem cells (MSCs). A phospho‐mutant mouse model shows that constitutive AMPKα1 activation prevents age‐related bone loss and promoted MSC osteogenic commitment with increased bone‐derived insulin‐like growth factor 1 (IGF‐1) secretion. Mechanistically, upregulation of IGF‐1 signalling by AMPKα1 depends on cAMP‐response element binding protein (CREB)‐mediated transcriptional regulation. Furthermore, the essential role of the AMPKα1/IGF‐1/CREB axis in promoting aged MSC osteogenic potential is confirmed using three‐dimensional (3D) culture systems. Taken together, these results can provide mechanistic insight into the protective effect of AMPKα1 against skeletal aging by promoting bone‐derived IGF‐1 secretion.

## Graphical abstract

Senile osteoporosis is the leading cause of bone loss and fragility fracture in the elderly population, but little is known to date about its aetiology. In this study, we identify AMP‐activated protein kinase alpha 1 (AMPKα1) as a critical factor regulating the senescence and lineage commitment of mesenchymal stem cells (MSCs). Constitutive AMPKα1 activation prevents age‐related bone loss, promotes MSC osteogenic commitment and increases bone‐derived insulin‐like growth factor 1 (IGF‐1) secretion. Mechanistically, CREB‐mediated transcriptional regulation is vital for IGF‐1 signalling activation by AMPKα1, and the CREB binding site in *Igf1* promoter is further located.

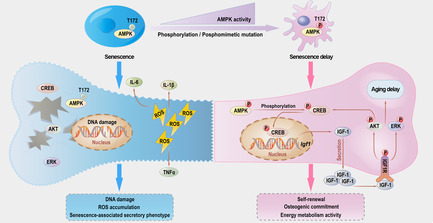

## 1 INTRODUCTION

Skeletal aging, also called senile osteoporosis (SOP), is characterized by age‐related bone loss and deterioration of bone microarchitecture.^1^, ^2^ By 2050, people >60 years old are predicted to number 1.5 billion, >20% of the total population worldwide.^3^ In this context, SOP and its complications distinctly increase the mortality of aged individuals and impose a huge financial burden on society. In contrast with the hyperactivity of bone resorption in postmenopausal osteoporosis, lack of bone formation is the main cause of SOP.^4^ Previous studies have shown that SOP is accompanied by inhibited mesenchymal stem cell (MSC) function, while multiple pathological factors, including intracellular reactive oxygen species (ROS) accumulation, deoxyribonucleic acid (DNA) damage, decreased metabolic activity and diminished osteogenic differentiation, are related to its aetiology.^5^ To develop novel and effective treatments for SOP, it is essential to understand which regulatory proteins help to maintain bone homeostasis in skeletal aging.

Adenosine monophosphate‐activated protein kinase (AMPK) is the major sensor and regulator of cellular‐energy metabolism.^6^ AMPK, which is a serine (Ser)/threonine (Thr) kinase complex, consists of α catalytic subunits (α1, α2), β scaffolding subunits (β1, β2) and γ regulatory subunits (γ1, γ2, γ3).^6^, ^7^ Phosphorylation of Thr172 (T172) in the α subunits by upstream signals is required for AMPK activation; subsequently, activated AMPK can further phosphorylate a wide range of downstream targets, such as acetyl coenzyme A (acetyl‐CoA) carboxylase (ACC), glucose transporter 4 (GLUT4), and cyclic adenosine monophosphate response element binding protein (CREB). Our previous studies found that AMPKα1 is the major isoform expressed in bone tissue and that its activation significantly promotes MSC osteogenic differentiation both in vitro and in vivo.^8^ Although the importance of AMPKα1 in bone remodelling is known, its role in skeletal aging is still debated.

Insulin‐like growth factor 1 (IGF‐1) is reported to be positively correlated with bone mass, and it is regarded as an independent predictor for the risk of osteoporosis.^9^ Notably, autocrine IGF‐1 in serum is secreted by the liver, whereas bone‐derived IGF‐1 deposited in bone matrix and marrow is secreted by skeletal cells, especially MSCs.^10^ Bone‐derived IGF‐1 acts in an autocrine/paracrine manner, showing a stronger effect on bone homeostasis than autocrine IGF‐1 does. Previous papers have demonstrated that matrix IGF‐1 helps to develop an osteogenic microenvironment and enhances osteogenesis of recruited MSCs.^11^ Emerging evidence again indicates that the IGF‐1 pool in bone matrix might be essential for new bone formation in the aging process.^12^ However, the regulatory mechanisms governing bone‐derived IGF‐1 expression during senescence are still not fully understood.

In this study, we found the aging of MSCs to be accompanied by obvious AMPKα1 inhibition. The results of our phospho‐mutant transgenic mouse model demonstrated that constitutive AMPKα1 activation protected against age‐related bone loss in vivo and delayed MSC aging in vitro with increased bone‐derived IGF‐1 secretion. Mechanistically, upregulation of IGF‐1 signalling by AMPKα1 was found to depend on CREB‐mediated transcriptional regulation. We confirmed this essential AMPKα1/IGF‐1/CREB axis in both a two‐dimensional (2D) cell model and a three‐dimensional (3D) culture environment.

## 2 MATERIALS AND METHODS

### 2.1 Mice

*Prkaa1‐T172D* ^*fl/fl*^ phospho‐mutant knock‐in mice were generated by GemPharmatech (Nanjing, China) from embryonic stem cells (ESCs) modified via homologous recombination. To express phospho‐mutant AMPKα1 specifically in MSCs, we crossed *Prkaa1‐T172D* ^*fl/fl*^ mice with *Prx1*‐*Cre* mice to generate conditionally AMPKα1 phospho‐mutant mice (AMPKα1^T172D^). All phospho‐mutant mice had a C57BL/6 genetic background. All mice were bred in a specific‐pathogen‐free (SPF) environment. All animal experiments were approved by the Animal Ethics Committee of Shanghai Ninth People's Hospital, Shanghai Jiao Tong University School of Medicine, Shanghai, China (Nos. HKDL‐2016‐221 and SH9H‐2022‐A1‐1). Due to oestrogen withdrawal, C57BL/6 female mice undergo a rapid decline in bone mass after maturity. To exclude putative influences of sex steroid hormone changes on the findings, male mice were chosen for this skeletal aging study.^13^

### 2.2 Cell culture

We obtained the cell line C3H10T1/2 from the American Type Culture Collection (ATCC; Manassas, VA, USA). Cells were cultured in α‐minimum essential medium (α‐MEM; GIBCO [ThermoFisher Scientific, Waltham, MA, USA]) with 10% fetal bovine serum (FBS) and 1% penicillin‐streptomycin solution (both GIBCO) at 37°C in a humidified atmosphere containing 5% CO_2_. To induce osteogenesis, we treated C3H10T1/2 cells with L‐ascorbic acid (50 μg/mL), β‐glycerophosphate (β‐GP; 10 mM), and bone morphogenetic protein 2 (BMP‐2; 100 ng/mL). To induce adipogenesis, C3H10T1/2 cells were treated with insulin (10 μg/mL), dexamethasone (1 μM), isobutylmethylxanthine (IBMX; 0.5 mM) and rosiglitazone (5 μM). The culture medium was changed every 2 days. We stained cells for ALP using an ALP Colour Development Kit (#C3206; Beyotime, Shanghai, China) after 14 days of osteogenic induction. Lipid droplets were stained using an Oil Red O Staining Kit (#C0157; Beyotime) after 7 days of adipogenic induction.

### 2.3 Isolation and expansion of primary mouse MSCs

We isolated primary mouse MSCs following a previous published study.^14^ These MSCs were cultured, serially passaged, and expanded using a MesenCult Expansion Kit (#05513; STEMCELL Technologies, Inc., Vancouver, BC, Canada) in vitro. Mouse MSCs in passage 2 were used in the experiments described below unless specifically emphasized otherwise. MSCs were probed for β‐galactosidase (β‐gal) expression using a commercial Senescence β‐Galactosidase Staining Kit (#C0602; Beyotime).

To induce osteogenesis, we cultured primary MSCs in α‐MEM supplemented with L‐ascorbic acid (50 μg/mL), β‐GP (10 mM), and dexamethasone (DXM; 10 nM). To induce adipogenesis, primary MSCs were cultured in high‐glucose Dulbecco's modified Eagle medium (DMEM; GIBCO) supplemented with insulin (10 μg/mL), DXM (1 μM), isobutylmethylxanthine (IBMX; 0.5 mM) and rosiglitazone (5 μM).

For the CFU assay, we plated 1 million nuclear MSCs per well in 12‐well plates in MSC expansion medium. After 14 days of cultivation, MSC‐derived colonies were stained using a Crystal Violet Staining Kit (#C0121; Beyotime). For the CFU‐ALP assay, we plated 0.5 million nuclear MSCs per well in 12‐well plates in MSC expansion medium. After 7 days, mouse MSCs were induced for osteogenesis as previously described. At Day 14, we stained the cells for ALP using the ALP Colour Development Kit. For the population‐doubling assay, the first passaged mouse MSCs were plated in 10‐cm plates (1 × 10^6^ cells). We changed the medium every 2 days and passaged MSCs at weekly intervals. MSCs were counted at each passage using an EVOS FL Imaging System.

### 2.4 Identification of MSC surface markers by flow cytometric analysis

Primary MSCs were isolated and serially passaged using MesenCult Expansion Kit in vitro. In passage 6, MSCs were collected and blocked with Mouse BD Fc Block™ (#553141; BD Pharmingen, USA) at 4°C for 5 min. Then, samples were incubated with the following antibodies: Rat IgG2b kappa Isotype Control‐FITC (1:200; #11‐4031‐82; Thermo Fisher, UK), Rat IgG2a kappa Isotype Control‐PE (1:200; #12‐4321‐80; Thermo Fisher, UK), CD106 Monoclonal Antibody‐PE (1:200; #12‐1061‐82; Thermo Fisher, UK), CD44 Monoclonal Antibody‐PE (1:200; #12‐0441‐82; Thermo Fisher, UK), CD29 Monoclonal Antibody‐PE (1:200; #12‐0291‐82; Thermo Fisher, UK), CD45 Monoclonal Antibody‐FITC (1:200; #11‐0451‐82; Thermo Fisher, UK), CD31 Monoclonal Antibody‐FITC (1:200; #11‐0311‐82; Thermo Fisher, UK), Ter‐119 Monoclonal Antibody‐FITC (1:200; #11‐5921‐82; Thermo Fisher, UK), CD11b Monoclonal Antibody‐FITC (1:200; #11‐0112‐82; Thermo Fisher, UK), in the dark 4°C for 30 min. The labelled MSCs were washed and analysed using flow cytometry (BD FACS Fortessa).

### 2.5 Real‐time quantitative polymerase chain reaction (RT‐qPCR) analysis

Total RNA was extracted from cells using TRIzol regent (#335904; Invitrogen Corp., Carlsbad, CA, USA), and RNA was reverse transcribed to complementary DNA (cDNA) using a GoScript RT Reagent Kit (#A5001; Promega Corp., Fitchburg, WI, USA). We performed RT‐qPCR using a QuantStudio 6 Flex RT‐qPCR System (Applied Biosystems, Foster City, CA, USA) with SYBR Green PCR Mix (#B21703; Bimake, Houston, TX, USA). All RT‐qPCR primers are listed in Table S1.

### 2.6 Immunoblotting

Cells were lysed with cell lysis buffer (#P0013, Beyotime) containing a phosphatase/protease inhibitor cocktail (1:100; #78441; Invitrogen). The protein concentration was quantified by BCA method, and equal amounts of total protein (25 μg) were loaded per well. Then, proteins were separated by sodium dodecyl sulfate polyacrylamide gel electrophoresis (SDS‐PAGE), transferred onto polyvinylidene fluoride (PVDF) membranes (MilliporeSigma, Burlington, MA, USA), blocked with 5% bovine serum albumin (BSA), and then immunoblotted with the indicated antibodies. We used an eBlot L1 Protein Transfer System (GenScript Corp., Jiangsu, China) for protein electrophoresis. The following antibodies were used in this study: anti‐AMPKα1 (1:1000); anti‐pAMPKα1 (1:1000); anti‐ACC (1:1000); anti‐pACC (1:1000); anti‐β‐actin (1:5000; Proteintech, Chicago, IL, USA); anti‐IGF‐1 (1:1000; Abcam, Cambridge, UK); anti‐IGF‐1R (1:1000; Proteintech); anti‐pIGF‐1R (1:1000); anti‐Akt (1:1000; Affinity Biosciences, Cincinnati, OH, USA); anti‐pAkt (1:1000; Affinity); anti‐ERK (1:1000); anti‐pERK (1:1000); anti‐CREB (1:1000); anti‐pCREB (1:1000); anti‐rabbit immunoglobulin G (IgG; 1:15000); and anti‐mouse IgG (1:15000; except where indicated otherwise, all antibodies were purchased from Cell Signalling Technology [CST], Danvers, MA, USA). Finally, we detected protein bands using an Odyssey fluorescence imaging system (LI‐COR Biosciences, Lincoln, NE, USA). The grayscale intensity of WB bands was quantitated with ImageJ software.

### 2.7 AMPK activity measurement

AMPK activity was quantitatively measured using an AMPK Assay Kit (#GMS50140.1; GemMed, USA). Briefly, per manufacturer's instructions, we collected cells treated with Reagent A and centrifuged them in 15‐mL tubes at 500 *g* for 3 min. The supernatant was then discarded. After adding Reagent B (300 μL), we transferred the mixture to a pre‐chilled 1.5‐mL microcentrifuge tube and incubated it at 4°C for 30 min. Next, we centrifuged samples at 16,000 *g* for 5 min to collect the supernatants for protein quantitation. Finally, the supernatants were mixed with kinase activity buffer, and absorbance was measured at 340 nm separately at the first and sixth minutes.

### 2.8 μCT analysis

Femurs and fifth lumbar vertebrae (L5) were harvested for high‐resolution μCT analysis (μCT 80, Scanco, Zurich, Switzerland) at a resolution of 10‐μm voxel size. Quantification of microarchitecture parameters were performed using Scanco software.

### 2.9 Histological analysis and immunostaining

Samples were fixed in 4% paraformaldehyde, decalcified in 10% ethylenediaminetetraacetic acid (EDTA), embedded in paraffin, sectioned at 4 μm, and finally processed with H&E or TRAP staining. We performed IHC using the antibody against pACC (1:200; CST). Samples were counterstained with haematoxylin (Sigma‐Aldrich [MilliporeSigma]), and images were obtained using a Pannoramic DESK P250 (3DHISTECH, Budapest, Hungary).

### 2.10 ELISA assay

Serum concentrations of P1NP and CTX‐1 were determined using a Mouse P1NP ELISA Kit (#E‐EL‐M0233) and a Mouse CTX‐1 ELISA Kit (#E‐EL‐M3023; both Elabscience, Houston, TX, USA) per the instructions provided. In addition, we determined concentrations of IGF‐1 in mouse bone marrow and cell supernatant using a Mouse IGF‐1 ELISA Kit (#MG100; R&D Systems, Inc., Minneapolis, MN, USA) per manufacturer's instructions.

### 2.11 Intracellular ROS measurement

Intracellular ROS levels were assessed using an ROS Assay Kit (#S0033; Beyotime). Briefly, we incubated samples with dichlorodihydrofluorescein diacetate (DCFH‐DA) working solution (10 μM) for 15 min prior to analysis, and we washed them three times in phosphate‐buffered saline (PBS). Finally, stained cells were mounted with Hoechst 33258 Working Solution (#C1017; Beyotime), and the samples were observed under a confocal scanning microscope (#TCS SP8; Leica, Wetzlar, Germany).

### 2.12 Immunofluorescence

Cells were fixed in pre‐chilled 4% paraformaldehyde for 15 min, treated with 0.5% Triton for 5 min, blocked with 10% goat serum at 4°C for 60 min, and incubated with primary antibodies at 4°C overnight. The next day, we washed the samples three times in tris‐buffered saline + Polysorbate 20 (TBST) and incubated them with fluorescent secondary antibodies at room temperature (RT) for 60 min. Finally, the nuclei were stained with 4′,6‐diamidino‐2‐phenylindole (DAPI) Working Solution (#C1005; Beyotime) for 10 min, and the Leica confocal scanning microscope was used to acquire images. The following antibodies were used: anti‐Ki‐67 (1:1000; Abcam), anti‐LAP2β (1:500; BD Biosciences, Franklin Lakes, NJ, USA), anti‐γH2A (1:500; GeneTex, Inc., Irvine, CA, USA), anti‐ALP (1:200; Santa Cruz Biotechnology, Dallas, TX, USA), anti‐mouse IgG (1:1000; CST) and anti‐rabbit IgG (1:1000; CST).

### 2.13 Metabolic analysis

We performed metabolic analysis using a Seahorse XFe96 Analyser (Agilent Technologies, Inc., Santa Clara, CA, USA) per the provided instructions. Briefly, primary MSCs (2 × 10^4^/well) were plated in XF‐96 culture plates 1 day before the experiment. In the oxygen consumption rate (OCR) assay, cells were treated with oligomycin (1 μM), carbonyl cyanide‐4‐(trifluoromethoxy) phenylhydrazone (FCCP; 2 μM), and rotenone (0.5 μM) + antimycin A (1.25 μM) as protocol. Then, we exported the oxidative‐phosphorylation parameters using the XF Mito Stress Test Report. In the extracellular‐acidification rate (ECAR) assay, cells were treated with glucose (10 mM), oligomycin (10 μM), and 2‐deoxy‐d‐glucose (2‐DG; 50 mM) as protocol. Then, we exported the glycolysis parameters using the XF Glyco Stress Test Report.

### 2.14 RNA sequencing

MSCs were isolated and expanded from 22‐month‐old AMPKα1^T172D^ mice and WT controls. At passage 2, we isolated total RNA from both groups for cDNA library preparation. Sequencing was performed following Illumina's (San Diego, CA, USA) standard protocol. We performed three independent replicates per group. KEGG and GO enrichment analyses and heatmap analysis were performed for all DEGs.

### 2.15 Cell line construction

We knocked down AMPKα1 using shRNA with lentivirus transfection following the provided instructions. Knockdown efficiency was evaluated using Western blot (WB). Similarly, we overexpressed phospho‐mutant AMPKα1 (T172D) via lentivirus transfection. Overexpression efficiency was evaluated using WB.

A CREB‐deficient cell line was generated using a clustered regularly interspaced short palindromic repeats (CRISPR)–CRISPR‐associated protein 9 (Cas9) lentivirus based on phospho‐mutant C3H10T1/2 cells and primary MSCs. We assessed CREB‐KO efficiency via WB. Lentivirus service was provided by Genomeditech (Shanghai, China). All primer sequences used in cell line construction are listed in Table S2.

### 2.16 Luciferase reporter assay

We constructed an *Igf1* full‐length promoter reporter and its truncated mutants using the pGL4.10 vector (Promega). A luciferase reporter assay was performed using the Dual‐Lumi II Luciferase Reporter Gene Assay Kit (#RG089; Beyotime) following the provided instructions. Briefly, we transfected cells in 96‐well plates with indicated vectors and reporter plasmids using Lipofectamine 3000 reagent (Thermo Fisher). Forty‐eight hours after transfection, cells were lysed in cell lysis buffer (#RG126; Beyotime), and luminescence signals were detected using a GloMax 20/20 Luminometer (Promega).

### 2.17 ChIP assay

For our ChIP assay, we used a SimpleChIP Plus Enzymatic Chromatin IP Kit (#9005; CST) per manufacturer's instructions. Briefly, cells were crosslinked using 1% formaldehyde at RT for 20 min. Then, samples were collected, washed with PBS, lysed in lysis buffer, and sonicated to produce ~400‐bp DNA fragments. Next, we immunoprecipitated these fragments with the indicated antibodies and finally pulled them down using magnetic beads. DNA purification columns from the ChIP Assay Kit were used for DNA purification. We assessed DNA sequence enrichment via RT‐qPCR. All primer sequences used in the ChIP assay are listed in Table S3.

### 2.18 3D culture system establishment

Primary MSCs from 22‐month‐old mice were used for bioprinting. Based on our previous studies, we performed 3D bioprinting using an EnvisionTEC 3D‐Bioplotter (EnvisionTEC, Gladbeck, Germany).^15^ First, we prepared the bioink as previously described. Sterile gelatin methacrylate (gelMA; 5% w/v^−1^), methacrylated hyaluronic acid (HAMA; 1% w/v^−1^), lithium phenyl‐2,4,6‐trimethylbenzoylphosphinate (LAP; 0.5% w/v^−1^), and type I collagen (0.18% w/v^−1^) were dissolved in PBS. We vigorously stirred the bioink in the dark at 37°C overnight to obtain a homogenous pre‐hydrogel bioink. Before loading the bioink with cells, we neutralized it with 1 M NaOH. Then, 50 μL of various primary‐MSC (passage 2) suspensions (1 × 10^7^ cells) was added to 1 mL prehydrogel solution and gently stirred in the dark to achieve uniformity. During bioprinting, we printed the MSC‐loaded bioink into a layer‐by‐layer lattice structure (10 × 10 × 3 mm; 10 layers). Fibre diameter was 300 μm, printing speed was 10 mm s^−1^, and nozzle temperature was 15°C. Next, 3D hydrogels were ultraviolet (UV) crosslinked for 60 s on the printing platform. After bioprinting, we cultured 3D cell models in osteogenic‐induction media with the indicated treatment. At Day 21, osteogenic outcomes of different groups were evaluated.

### 2.19 Statistics

All data are presented as mean ± standard deviation (SD), and all results are expressed as bar plots with individual data points. Statistical analysis is performed using Prism Version 9 Software (GraphPad, CA, USA). Significant comparisons between two groups are analysed using unpaired, two‐tailed Student's *t* tests. Significant comparisons of three or more groups are analysed using one‐way analysis of variance (ANOVA) with Tukey post‐hoc test. For conditions which are affected by two variables, two‐way ANOVA with Benjamini, Krieger and Yekutieli post‐hoc test is used. *p* < 0.05 is considered statistically significantly different.

## 3 RESULTS

### 3.1 MSC aging and lineage commitment are accompanied by changes in AMPKα1 activity

**FIGURE 1:**
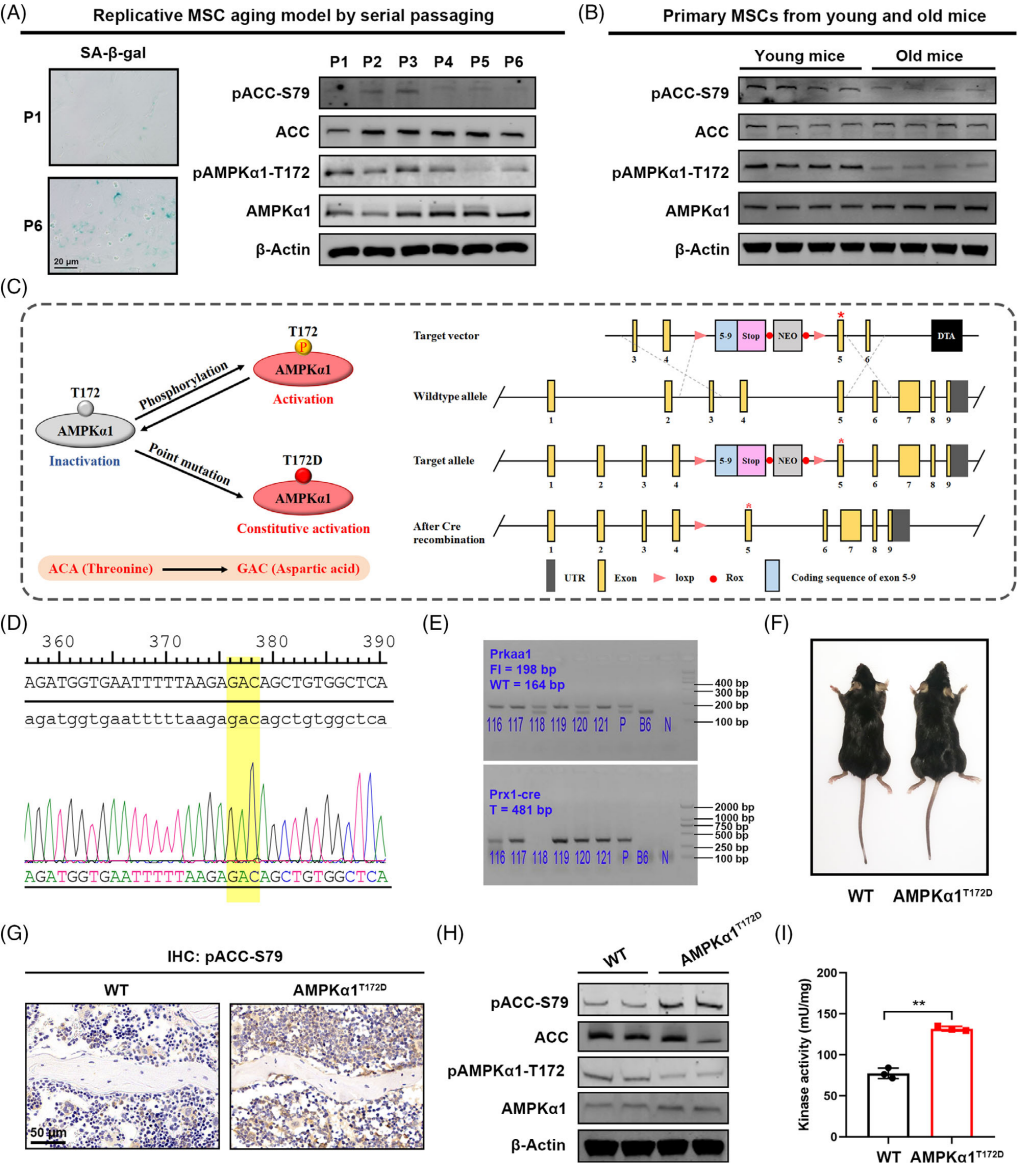
Generation of phospho‐mutant AMPKα1^T172D^ mice for constitutive AMPK activation. (A) Representative SA‐β‐gal staining images of primary mouse mesenchymal stem cells (MSCs) with serial passaging. Immunoblotting showing pAMPKα1‐T172 and pACC‐S79 expression during in vitro passaging (P1–P6). (B) Immunoblotting showing pAMPKα1‐T172 and pACC‐S79 expression in primary MSCs from aged mice (22 months) as compared to those from young mice (3 months). (C) Schematic illustration of the strategy to generate AMPKα1^T172D^ mice. To constitutively activate AMPK, we introduce a phospho‐mimetic mutation into AMPKα1 by substituted Thr172 with aspartic acid (T172D). After *Cre* recombination, the sequence between *loxp* sites was deleted, and phospho‐mutant AMPKα1 was expressed. (D) Sanger sequencing validated successful phospho‐mimetic mutation in primary MSCs. (E) Genotyping of offspring mice. P: plasmid DNA as positive control; B6: B6 genomic DNA as negative control; N: H_2_O as blank control. (F) Representative photographs of AMPKα1^T172D^ mice and WT littermate controls. (G) IHC staining showing pACC‐S79 expression in bone tissues. (H) Immunoblotting showing pACC‐S79, ACC, pAMPKα1‐T172 and AMPKα1 expression in primary MSCs from AMPKα1^T172D^ mice and WT controls. (I) Measurement of AMPK activity in primary MSCs from AMPKα1^T172D^ mice and WT controls (*n* = 3). Results are presented as bar plots with all data points. **p < 0.01.

Physiological AMPK activation mainly involves specific phosphorylation of the AMPK catalytic α1 subunit at T172 (pAMPKα1‐T172), after which the activated AMPKα1 mediates upstream direct phosphorylation of ACC at the Ser79 (S79) residue (pACC‐S79; Figure S1). So far, a range of studies have reported AMPKα1 activity to be positively correlated with the ratios of pAMPKα1‐T172/AMPKα1 and pACC‐S79/ACC.^16^ To explore the role of AMPKα1 in MSC aging, a replicative MSC aging model was first established by serial passaging in vitro, and we examined the expression profile of pAMPKα1‐T172 and pACC‐S79 during this process. MSC purity was determined by flow cytometry at passage 6: there was high expression of CD29 (84.6%), CD44 (99.7%) and CD106 (22.0%), but negative expression of CD45 (2.11%), Ter‐119 (0.97%), CD31 (1.73%) and CD11b (2.19%) (Figure S2). Notably, we further found that the ratios of pAMPKα1‐T172/AMPKα1 and pACC‐S79/ACC in MSCs significantly decreased after three passages, concurrent with an increase of positive senescence‐associated β‐galactosidase (SA‐β‐gal) cells (Figure 1A). The quantification of WB results was shown in Figure S3A. Next, we isolated primary MSCs separately from young wild‐type (WT) mice (3 months) and old mice (22 months) for immunoblotting to study the changes of AMPKα1 activation between young and old individuals (Figure 1B). Correspondingly, we detected decreased pAMPKα1‐T172/AMPKα1 and pACC‐S79/ACC ratios in old mice. The quantification of WB results was shown in Figure S3B. These observations indicated that mouse MSC aging was accompanied with AMPKα1 inhibition.

MSC aging is marked by a switch from osteogenic to adipogenic commitment. Dysregulation of this osteo‐adipogenic balance is a hallmark of MSC senescence.^17^ To explore changes in AMPKα1 activity in the context of adipo‐osteogenic balance, we examined the ratios of pAMPKα1‐T172/AMPKα1 and pACC‐S79/ACC with MSC differentiation. As detected by immunoblotting, expression of both showed a gradual increase during osteogenic induction but an obvious decrease during adipogenic induction (Figure S4). In other words, our results showed that AMPKα1 activity in MSCs was correlated positively with osteogenic commitment and negatively with adipogenic commitment. Therefore, we speculated that AMPKα1 activation might be involved in MSC aging regulation.

### 3.2 Generation and characteristics of phospho‐mutant AMPKα1^T172D^ mice

Since the average mouse lifespan is >12 months, experiments on MSC senescence require prolonged periods of observation and intervention. However, our previously published studies showed that widely used AMPK agonists such as metformin and 5‐aminoimidazole‐4‐carboxamide ribonucleotide (AICAR) failed to continuously activate AMPK for >24 h.^18^ Therefore, we aimed to develop a transgenic‐mouse model with constitutive AMPKα1 activation to study skeletal aging in vivo. To this end, we introduced a phospho‐mimetic mutation into AMPKα1 by substituting Thr172 with an aspartic acid (hereafter designated T172D) to produce *Prkaa1*–*T172D* ^*fl/fl*^ knock‐in mice. Furthermore, to specifically express phospho‐mutant AMPKα1 in MSCs, we crossed *Prkaa1‐T172D* ^*fl/fl*^ mice with *Prx1*‐*Cre* mice to generate conditionally AMPKα1 phospho‐mutant (AMPKα1^T172D^) mice (Figure 1C). Sanger sequencing analysis confirmed the successful introduction of phospho‐mimetic mutations (Figure 1D), while genotyping confirmed successful insertion of target DNA fragments into the mice's genomic DNA (Figure 1E).

As shown in gross photograph, AMPKα1^T172D^ mice exhibited no obvious changes in body size compared with their WT littermate controls (Figure 1F). Notably, both immunohistochemical (IHC) staining and immunoblotting revealed obviously higher expression of pACC‐S79 in primary MSCs from AMPKα1^T172D^ mice (Figure 1G,H). The quantification of WB results was shown in Figure S3C. Consistently, more‐direct evidence came from the AMPK activity assay, in which AMPK isolated from phospho‐mutant MSCs showed higher kinase activity than that isolated from controls (Figure 1I). These results confirmed efficient activation of AMPKα1 in primary MSCs from AMPKα1^T172D^ mice.

### 3.3 Constitutively activated AMPKα1 protects against age‐related bone loss in mice

**FIGURE 2:**
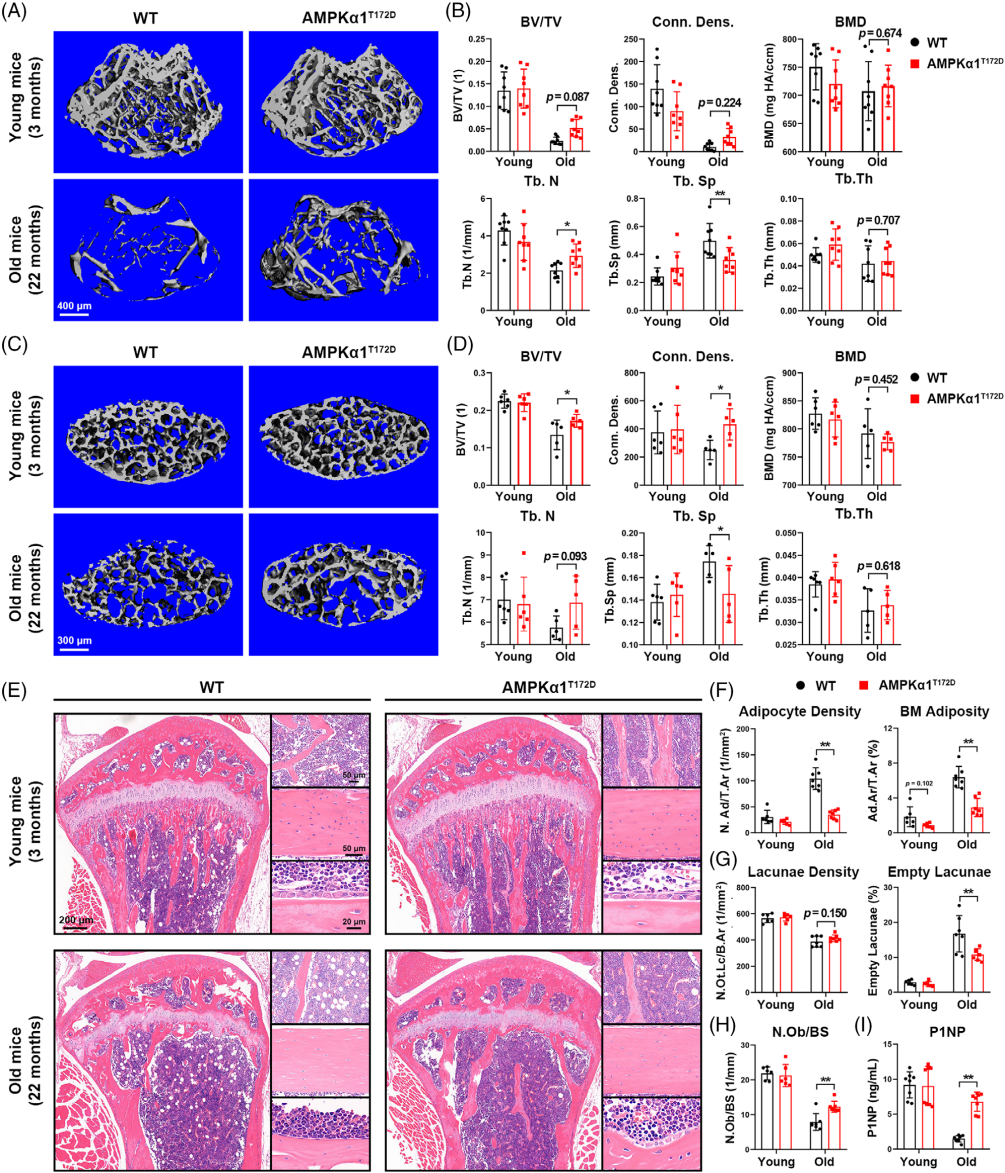
Constitutively activated AMPKα1 protects against age‐related bone loss in mice. (A) 3D μCT images of femoral trabecular bones from AMPKα1^T172D^ mice and WT controls (*n* = 8). (B) Quantitative μCT morphometric analysis of trabecular bones. (C) 3D μCT images of vertebral bodies (L5) from AMPKα1^T172D^ mice and WT controls (*n* = 5–6). (D) Quantitative μCT morphometric analysis of vertebral bodies. (E) Representative H&E staining images of tibia from AMPKα1^T172D^ mice and WT controls (*n* = 6–7). Left panel: a panoramic view of the proximal tibia; Right upper panel: a zoom‐in view of adipocytes in bone marrow beneath the growth plate; Right middle panel: a zoom‐in view of lacunae in tibia cortical bone; Right lower panel: a zoom‐in view of osteoblasts lining bony trabeculae. (F) Quantitative morphometric analysis of adipocyte density (adipocyte number per total area, N.Ad/T.Ar) and BM adiposity (adipocyte area per total area, Ad.Ar/T.Ar). BM = bone marrow. (G) Quantitative morphometric analysis of lacunae density (lacunae number with/without osteocyte per bone area, N.Ot.Lc/B.Ar) and empty lacunae ratio. (H) Quantitative morphometric analysis of osteoblast number per bone surface (N.Ob/BS). (I) Serum P1NP concentrations of AMPKα1^T172D^ mice and WT controls (*n* = 8). Results are presented as bar plots with all data points. *p < 0.05; **p < 0.01.

Skeletal aging is characterized by age‐related bone loss, bone marrow adiposity and osteogenic cell diminishment. To elucidate the role of AMPKα1 activation in regulating bone accrual and age‐related bone loss, we analysed bone samples from AMPKα1^T172D^ mice and WT controls of different ages. Micro‐computed tomographic (μCT) analysis of both femoral trabecular bones and L5 vertebrae showed that bone microarchitecture parameters did not differ significantly between AMPKα1^T172D^ mice and WT controls in the adult stage (3 months) even though mutated AMPKα1 had been expressed from birth. This indicated that constitutive AMPKα1 activation barely affected bone accrual and remodelling during development. In contrast, bone volume per tissue volume (BV/TV), connectivity density (Conn.Dens.), and number of trabeculae (Tb.N) were appreciably increased in aged AMPKα1^T172D^ mice compared with WT controls (22 months); meanwhile, trabecular spacing (Tb.Sp) was decreased as indicated by μCT analysis (Figure 2A–D). No statistically significant difference in trabecular thickness (Tb.Th), bone mineral density (BMD), or cortical‐microarchitecture parameters was noted (Figure S5A,B).

Bone marrow adiposity and diminished number of osteocytes are hallmarks of skeletal aging.^14^, ^19^ Bone histomorphometry based on haematoxylin and eosin (H&E) staining further confirmed our μCT findings. No obvious difference was observed between AMPKα1^T172D^ mice and WT controls during the adult stage (3 months). However, more trabeculae and diminished adipose tissues were found within the bone marrow cavities of aged AMPKα1^T172D^ mice (Figure 2E). Both the adipocyte density (N.Ad/T.Ar) and the bone marrow adiposity parameter (Ad.Ar/T.Ar) were markedly decreased by constitutive AMPKα1 activation in aged mice (Figure 2F). Even though constitutive AMPKα1 activation failed to rescue the age‐induced reduction of lacunae density (N.Ot.Lc/B.Ar), osteocyte death was prevented to a marked extent, as indicated by the decreased empty lacunae ratio in aged AMPKα1^T172D^ mice (Figure 2G). The number of osteoblasts per unit bone surface (N.Ob/BS) and the level of serum procollagen type I intact N‐terminal propeptide (P1NP; a bone formation marker) were also increased in aged AMPKα1^T172D^ mice (Figure 2H,I). No significant difference in osteoclastogenic parameters was noted in aged mice (Figure S5C–E). Collectively, these results indicated that constitutive AMPKα1 activation could enhance bone formation and MSC osteogenic commitment in aging process.

### 3.4 Constitutively activated AMPKα1 delays primary MSC aging

**FIGURE 3:**
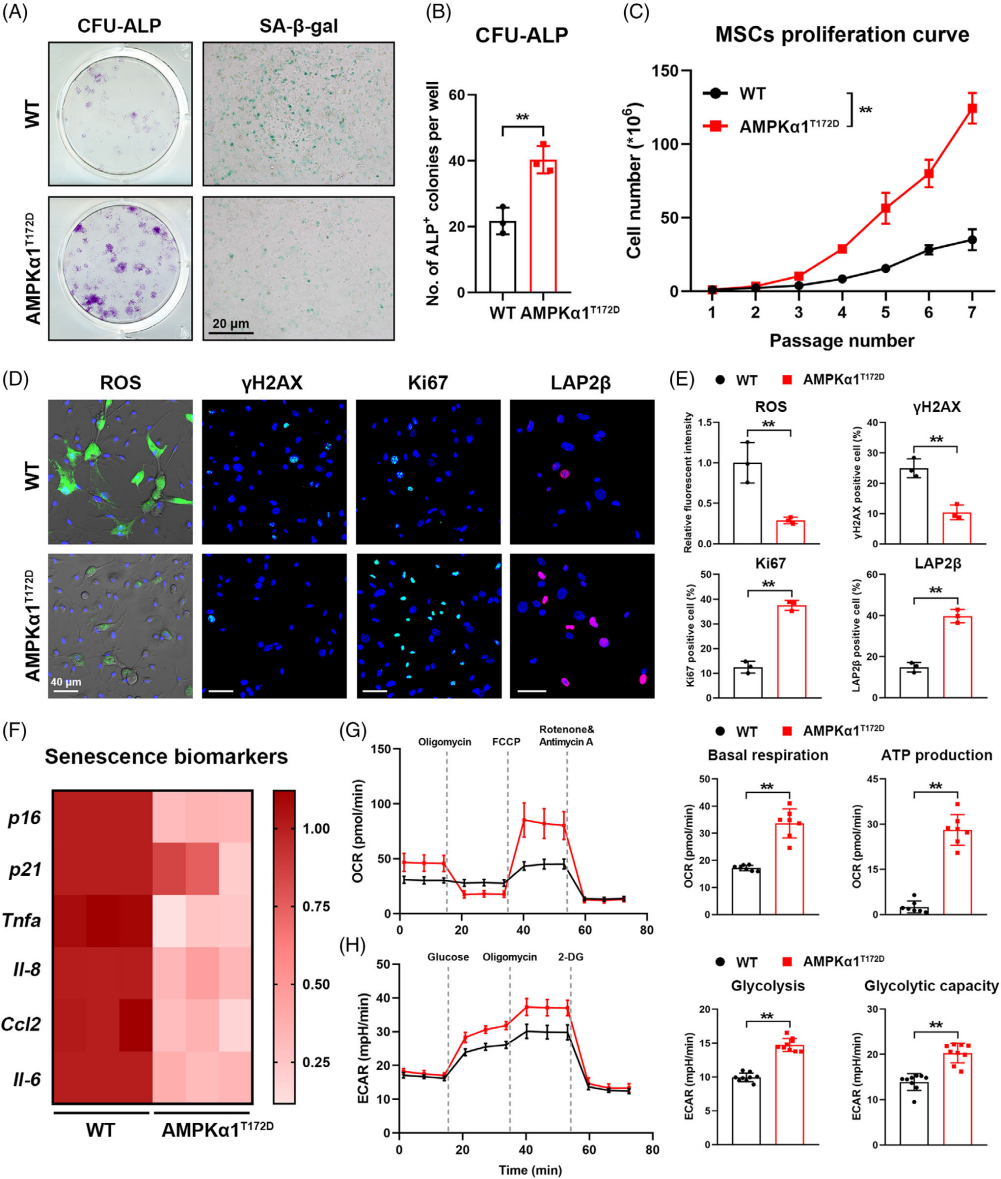
Constitutively activated AMPKα1 delays primary mesenchymal stem cell (MSC) aging. (A) Representative images of CFU‐ALP assay and SA‐β‐gal staining assay of primary MSCs from AMPKα1^T172D^ mice and WT controls at 22 months. (B) Quantification of ALP^+^ colonies (*n* = 3). (C) In vitro population doublings of MSCs from AMPKα1^T172D^ mice and WT controls at 22 months (*n* = 3). (D) Fluorescent staining of reactive oxygen species (ROS), γH2AX, Ki‐67 and LAP2β in MSCs from AMPKα1^T172D^ mice and WT controls at 22 months. (E) Quantification of intracellular ROS level based on fluorescent images. Quantitative analysis of Ki6‐7^+^, γH2AX^+^ and LAP2β^+^ frequencies in primary MSCs based on fluorescent images (*n* = 3). (F) Expression of senescence biomarkers (*p16*, *p21*, *Tnfa*, *Il‐8*, *Ccl2* and *Il‐6*) as detected by RT‐qPCR (*n* = 3). (G, H) Metabolic analysis (OCR and ECAR) of primary MSCs from AMPKα1^T172D^ mice and WT controls at 22 months (*n* = 7 for Mito stress test; *n* = 9 for Glycolysis stress test). Quantification of basal respiration, adenosine triphosphate (ATP) production, glycolysis, and glycolytic capacity. Results are presented as bar plots with all data points. **p < 0.01.

To explore the role of AMPKα1 activation in modulating MSC aging, we isolated aged primary mouse MSCs (22 months) for a colony forming unit‐alkaline phosphatase (CFU‐ALP) assay and SA‐β‐gal staining. Consistent with in vivo data, the aged phospho‐mutant MSCs exhibited higher self‐renewal and ALP^+^ colony formation capacity with less SA‐β‐gal expression compared with WT controls (Figure 3A,B). In terms of MSC replication capacity, primary MSCs from aged AMPKα1^T172D^ mice also displayed accelerated cell replication in vitro (Figure 3C).

Cell senescence is always accompanied by a serial change of biomarkers, such as intracellular ROS, γH2AX (a genomic‐DNA damage marker), Ki‐67 (a self‐renewal capacity marker), and lamina‐associated polypeptide 2 beta (LAP2β; a genomic‐DNA stability marker). Although primary MSC cultures from aged AMPKα1^T172D^ mice had relatively more Ki‐67^+^ and LAP2β^+^ cells than WT controls, we detected fewer γH2AX^+^ cells and reduced intracellular ROS levels in phospho‐mutant MSCs (Figure 3D,E). Senescent MSCs attempt to express high levels of aging‐associated markers, such as *p16* and *p21*, and a wide range of inflammatory cytokines. This secretome change is known as the senescence‐associated secretion phenotype (SASP).^20^ MSCs from aged AMPKα1^T172D^ mice exhibited significantly lower expression of *p16*, *p21*, and SASP biomarkers than WT controls (Figure 3F). Inhibited metabolic activity is another hallmark of cell senescence.^21^ According to Seahorse XF Analyser stress test reports, constitutively activated AMPKα1 enhanced cellular‐energy metabolic activity in aged MSCs, as evidenced by its promotion of not only mitochondrial respiration but also glycolysis (Figure 3G,H; Figure S6). These results indicated that constitutively activated AMPKα1 delayed MSC aging by promoting self‐renewal capacity, mitigating genomic‐DNA damage, and reprogramming metabolism.

### 3.5 Constitutively activated AMPKα1 promotes bone‐derived IGF‐1 production and activates IGF‐1 signalling pathway

**FIGURE 4:**
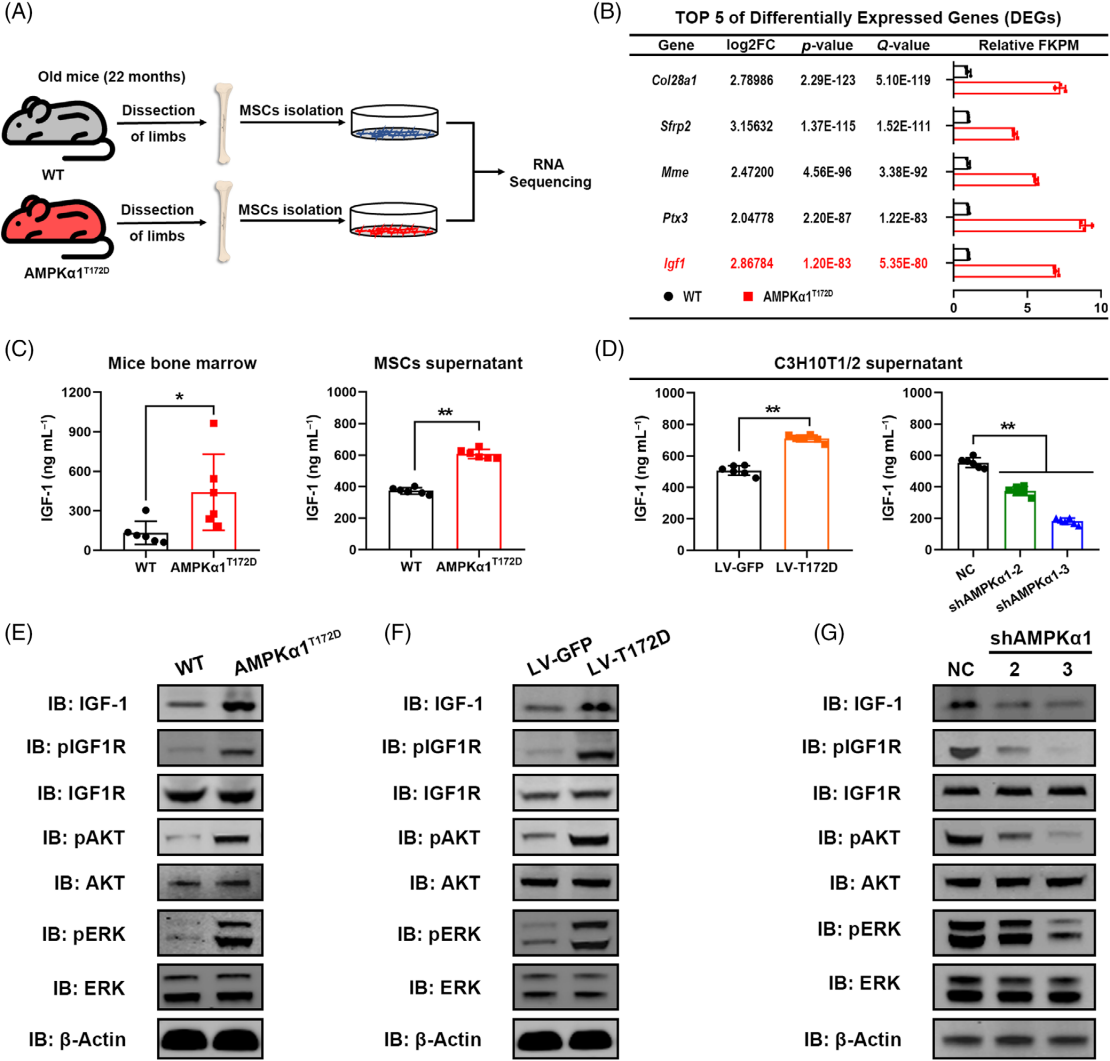
Constitutively activated AMPKα1 promotes bone‐derived IGF‐1 production and activates IGF‐1 signalling pathway. (A) Schematic illustration of experimental design of sample preparation for RNA sequencing. (B) Top 5 differentially expressed genes (DEGs) in sequencing. FPKM = Fragments Per Kilobase Million. (C) ELISA showing IGF‐1 protein concentrations in mouse bone marrow and primary‐mesenchymal stem cell (MSC) supernatant (*n* = 6). (D) ELISA showing IGF‐1 protein concentrations in C3H10T1/2 cell supernatant (*n* = 6). (E) Effects of constitutively activated AMPKα1 on IGF‐1 signalling pathway in primary MSCs. (F, G) Effects of AMPKα1 constitutive activation or AMPKα1 knockdown on IGF‐1 signalling pathway in C3H10T1/2 cells. Results are presented as bar plots with all data points. *p < 0.05; **p < 0.01.

To further investigate the molecular mechanism underlying the protective effect of AMPKα1 activation, we performed ribonucleic acid (RNA) sequencing to identify differentially expressed genes (DEGs) between aged AMPKα1^T172D^ mice and WT controls (Figure 4A). A total of 287 DEGs (fold change [FC] > 2; *p* < 0.05) were identified; 200 of the DEGs were upregulated, the other 87 downregulated (Figure S7). The top five most significant DEGs in sequencing were listed in Figure 4B (*p*‐values in ascending order). Collagen type XXVIII alpha 1 (*Col28a1*) and membrane metalloendopeptidase (*Mme*) are mainly involved in extracellular matrix metabolism^22^, ^23^; the frizzled‐related protein 2 (*Sfrp2*) is a Wnt signalling inhibitor and long pentraxin 3 (*Ptx3*) is an innate immune modulator.^24^, ^25^ By contrast, IGF‐1 is well‐acknowledged to significantly participate in cell aging and MSC commitment.^12^, ^26^ Therefore, we speculated that IGF‐1 derived from MSCs might be essential to the protective effect of AMPKα1 activation.

To verify this hypothesis, we first assessed bone‐derived IGF‐1 expression levels in vivo and in vitro. In accordance with RNA sequencing data, aged AMPKα1^T172D^ mice had higher IGF‐1 protein concentrations in bone marrow as detected by enzyme‐linked immunosorbent assay (ELISA; Figure 4C). In addition, primary MSCs from aged AMPKα1^T172D^ mice also showed higher IGF‐1 expression both in messenger RNA (mRNA) and protein levels than those from WT controls (Figure 4C; Figure S8A). To further explore the relationship between AMPKα1 activity and bone‐derived IGF‐1 expression, we next established a short‐hairpin RNA (shRNA)‐mediated AMPKα1 knockdown cell line (hereafter designated shAMPKα1, with negative control [NC]) and a T172D phospho‐mutant AMPKα1 cell line (hereafter designated LV‐T172D, with LV‐GFP as control) using C3H10T1/2 cells. Knockdown and phospho‐mutant efficiencies were confirmed by immunoblotting (Figure S9). Consistent with primary MSCs, IGF‐1 secretion in C3H10T1/2 cells was elevated by T172D phospho‐mimetic mutation but decreased by AMPKα1 knockdown (Figure 4D; Figure S8B).

IGF‐1 is a secreted peptide that functions by binding to its receptor and activating downstream signalling pathways. Therefore, we attempted to clarify whether AMPKα1 could activate IGF‐1 downstream signalling pathways. As shown by immunoblotting, AMPKα1 activation stimulated the phosphorylation of IGF‐1 receptor (IGF1R), protein kinase B (Akt), and extracellular signal‐regulated kinase (ERK) in primary MSCs (Figure 4E). Similarly, LV‐T172D cells also showed a higher activation level of IGF‐1 signalling (Figure 4F). In contrast, phosphorylation of IGF‐1R, Akt and ERK was remarkably inhibited after AMPKα1 knockdown in C3H10T1/2 cells (Figure 4G). The quantification of WB results was shown in Figure S10. In summary, these results demonstrated that constitutively activated AMPKα1 promoted bone‐derived IGF‐1 secretion and activated IGF‐1 signalling pathway both in vivo and in vitro.

### 3.6 IGF‐1 signalling pathway is vital to AMPKα1 modulation of aging and lineage commitment

**FIGURE 5:**
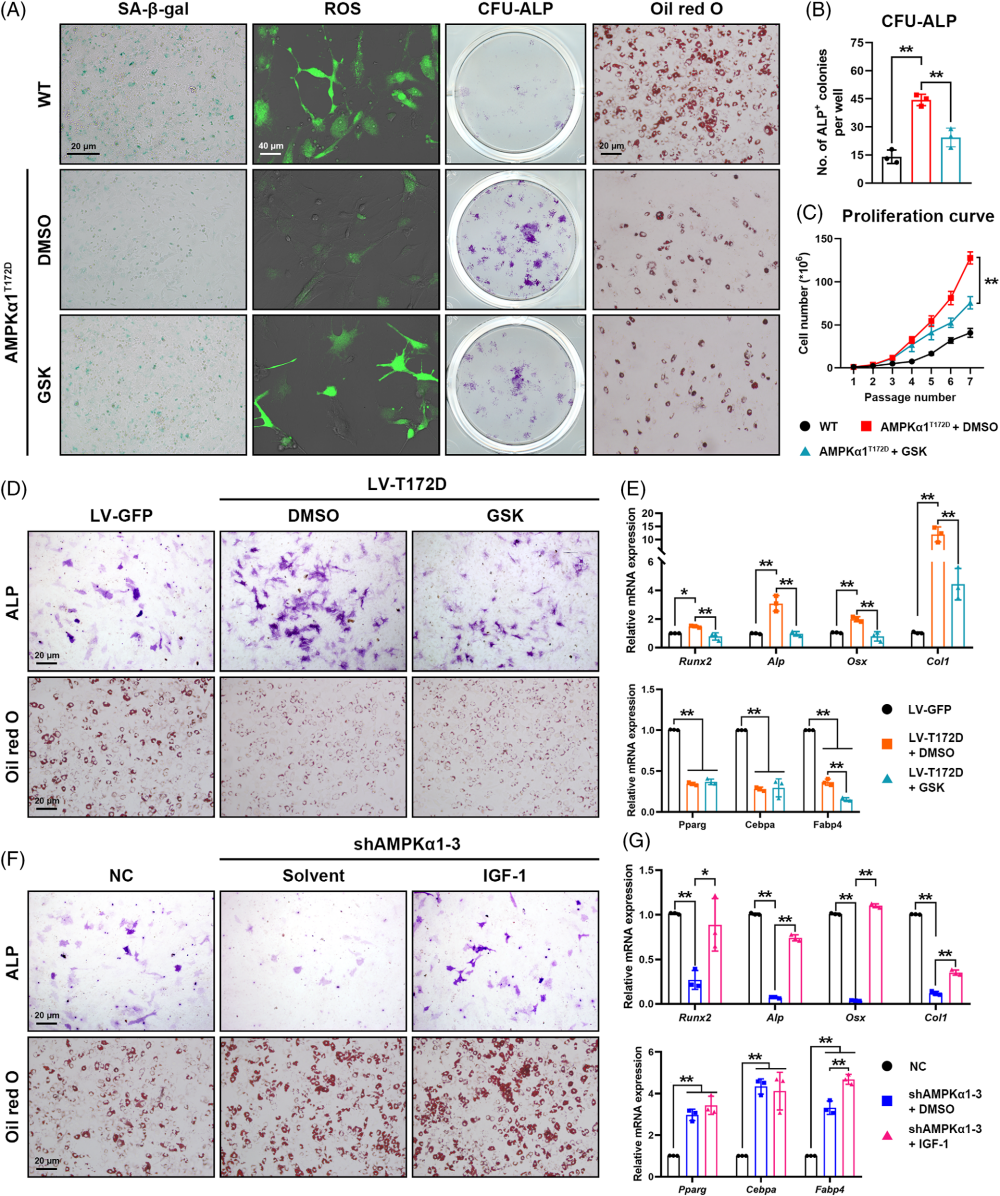
IGF‐1 signalling pathway is vital to AMPKα1 modulation of aging and lineage commitment. (A) Effects of GSK1838705A (GSK; a specific IGF‐1R inhibitor) to antagonize IGF‐1 signalling on SA‐β‐gal expression, intracellular reactive oxygen species accumulation, ALP^+^ colony formation, and lineage commitment in 22‐month‐old mesenchymal stem cells (MSCs) with constitutive AMPKα1 activation. DMSO was used as the solvent control. (B) Quantification of ALP^+^ colonies in CFU‐ALP assay (*n* = 3). (C) Effects of GSK on in vitro population doublings of MSCs with constitutive AMPKα1 activation (*n* = 3). (D) AMPKα1 was constitutively activated in C3H10T1/2 cells (LV‐T172D). Cell differentiation was assessed 7 days after adipogenic induction by Oil Red O staining or 14 days after osteogenic induction by ALP staining (*n* = 3). (E) Effects of GSK on the expression of osteogenic (*Runx2*, *Alp*, *Osx*, and *Col1*) and adipogenic (*Pparg*, *Cebpa* and *Fabp4*) markers as assessed by RT‐qPCR in C3H10T1/2 cells with constitutive AMPKα1 activation (*n* = 3). (F) AMPKα1 was knocked down in C3H10T1/2 cells (shAMPKα1‐3). Cell differentiation was assessed 7 days after adipogenic induction by Oil Red O staining or 14 days after osteogenic induction by ALP staining (*n* = 3). (G) Effects of exogenous IGF‐1 on expression of osteogenic (*Runx2*, *Alp*, *Osx* and *Col1*) and adipogenic (*Pparg*, *Cebpa* and *Fabp4*) markers as assessed by RT‐qPCR in C3H10T1/2 cells with AMPKα1 knockdown (*n* = 3). Results are presented as bar plots with all data points. *p < 0.05; **p < 0.01.

IGF‐1 is one of the most abundant growth factors deposited in bone marrow and matrix.^27^, ^28^, ^29^ Bone‐derived IGF‐1 has been acknowledged to contribute to the maintenance of skeletal homeostasis and bone mass during skeletal senescence.^12^ To investigate the role of bone‐derived IGF‐1 in AMPKα1 modulation of aging and lineage commitment, we treated aged phospho‐mutant MSCs with GSK1838705A (GSK; a specific IGF‐1R inhibitor) to block IGF‐1 signalling, using dimethyl sulfoxide (DMSO) as the solvent control. Notably, inhibition of IGF‐1 signalling by GSK efficiently counteracted AMPKα1‐mediated protection against MSC aging. SA‐β‐gal and intracellular ROS accumulation elevated again after GSK treatment in primary MSCs from 22‐month‐old AMPKα1^T172D^ mice (Figure 5A). Furthermore, GSK treatment also significantly inhibited AMPKα1^T172D^ MSC self‐renewal, replication, and ALP^+^ colony formation capacity along with an obvious arrest in the cell population doubling time (Figure 5B,C). In terms of lineage commitment, GSK treatment impaired AMPKα1‐mediated osteogenic potential but did not affect adipogenic differentiation, as indicated by parallel downregulation of osteogenic markers (*Runx2*, *Alp*, *Osx* and *Col1*) with adipogenic markers (*Pparg*, *Cebpa* and *Fabp4*) unchanged (Figure 5A; Figure S11).

Next, we reconfirmed our findings from MSCs in C3H10T1/2 cell line by treating LV‐T172D cells with GSK. Consistent with the primary MSC results, IGF‐1 signalling inhibition by GSK downregulated osteogenic‐gene expression and attenuated osteogenic differentiation induced by T172D phospho‐mutation, with only minimal effect on adipogenic differentiation (Figure 5D,E). We also investigated the rescuing effect of IGF‐1 activation on lineage commitment in the AMPKα1 knockdown C3H10T1/2 cell line. Exogenous IGF‐1 significantly rescued the inhibited osteogenic commitment of shAMPKα1 cells, as evidenced by increased ALP expression and upregulated osteogenic‐marker transcripts (Figure 5F,G; Figure S12). These results therefore suggested the vital role of IGF‐1 in AMPKα1 modulation of aging and lineage commitment.

### 3.7 AMPKα1 promotion of bone‐derived IGF‐1 secretion depends on CREB‐mediated transcriptional regulation

**FIGURE 6:**
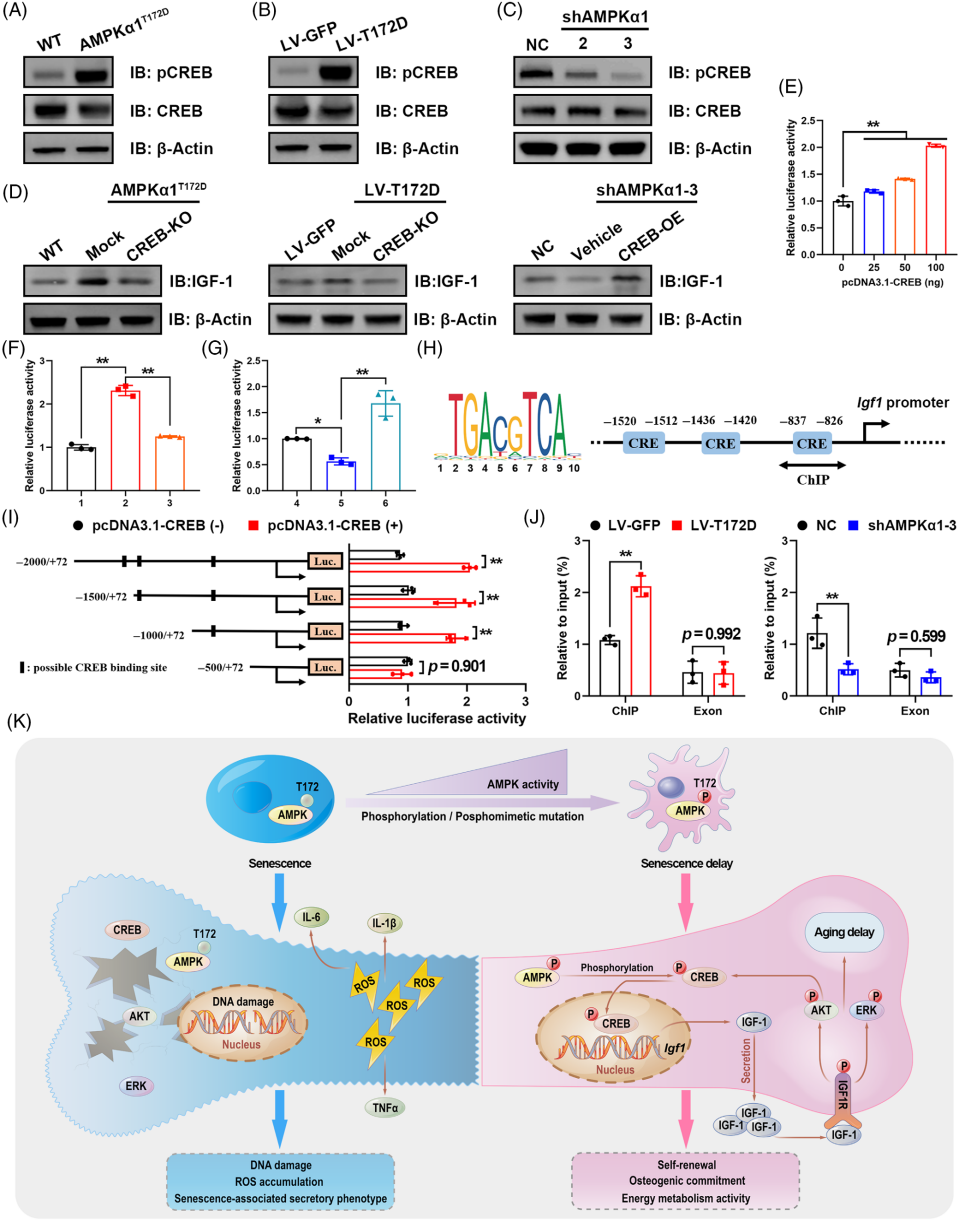
AMPKα1 promotion of bone‐derived IGF‐1 secretion depends on CREB‐mediated transcriptional regulation. (A) Protein expression of CREB in primary mesenchymal stem cells (MSCs) from AMPKα1^T172D^ mice and WT controls. (B, C) Protein expression of CREB in C3H10T1/2 cells with AMPKα1 constitutive activation or AMPKα1 knockdown. (D) Effects of CREB‐KO or overexpression on IGF‐1 protein expression in MSCs or C3H10T1/2 cells. (E) CREB‐enhanced *Igf1* promoter transcriptional activity as assessed by dual‐luciferase reporter assays in C3H10T1/2 cells (*n* = 3). (F, G) *Igf1* promoter transcriptional activity in indicated cell lines was assessed using dual‐luciferase reporter assays (*n* = 3). 1: LV‐GFP; 2: LV‐T172D + Mock; 3: LV‐T172D + CREB‐KO; 4: NC; 5: shAMPKα1‐3 + Vehicle; 6: shAMPKα1‐3 + CREB‐OE. (H) Predicted binding sites of CREB on the *Igf1* promoter. (I) Dual‐luciferase reporter assays were performed on C3H10T1/2 cells with the *Igf1* full‐length promoter reporter and its truncated mutants (*n* = 3). (J) ChIP analysis of CREB binding to the *Igf1* promoter (−837 to −826 bp) and exon regions (*n* = 3). (K) Schematic illustration of the proposed molecular mechanism by which constitutively activated AMPKα1 protects against MSC aging by promoting autocrine IGF‐1 secretion. CREB‐mediated transcriptional regulation is essential for this process. Results are presented as bar plots with all data points. *p < 0.05; **p < 0.01.

CREB is a major phosphorylation target protein of AMPK. Generally, AMPK directly phosphorylates CREB at Ser133 and drives its nuclear translocation for transcriptional regulation.^30^ Therefore, we speculated that CREB may be essential to bone‐derived IGF‐1 secretion promoted by AMPKα1 activation. To test this hypothesis, we first assessed changes in CREB phosphorylation under AMPKα1 influence. As expected, AMPKα1 activation significantly enhanced CREB phosphorylation in both primary MSCs and LV‐T172D C3H10T1/2 cells, whereas AMPKα1 knockdown obviously diminished pCREB expression (Figure 6A–C). The quantification of WB results was shown in Figure S13A. Next, we knocked out CREB protein in phospho‐mutant cells using a lentivirus‐mediated CRISPR‐Cas9 system (CREB‐KO, with mock as a control) while also overexpressing CREB protein in shAMPKα1 cells using a pcDNA3.1 expression plasmid (CREB‐OE, with vehicle as a control). Knockout (KO) and overexpression efficiencies were assessed via immunoblotting (Figure S14). Notably, CREB‐KO partially counteracted the increased IGF‐1 expression induced by AMPKα1 activation, whereas CREB overexpression rescued the decrease in IGF‐1 secretion due to AMPKα1 knockdown (Figure 6D; Figure S15A). The quantification of WB results was shown in Figure S13B. The above results indicated that regulation of bone‐derived IGF‐1 secretion by AMPKα1 depended on CREB.

To clarify the regulatory mechanism of CREB in *Igf1* gene transcription, we assessed *Igf1* promoter (−2000 to +72 bp) activity using dual‐luciferase reporter assays. Remarkably, exogenous CREB stimulated *Igf1* promoter‐driven luciferase expression in a dose‐dependent manner (Figure 6E). Furthermore, consistent with the gene's expression profile, CREB‐KO could cancel the increase in *Igf1* promoter activity due to AMPKα1 activation, and CREB overexpression could reverse the decrease in *Igf1* promoter activity due to AMPKα1 knockdown (Figure 6F,G; Figure S15B).

Next, bioinformatic analysis predicted three possible binding sites of CREB on the *Igf1* promoter (Figure 6H). To further identify the actual CREB binding site, we used the *Igf1* full‐length promoter reporter and its truncated mutants in dual‐luciferase reporter assays with CREB stimulation. The results confirmed that the actual binding site was about −830 bp (Figure 6I). Chromatin immunoprecipitation (ChIP) assays also confirmed the binding of CREB to this region on the *Igf1* promoter. Notably, constitutively activated AMPKα1 significantly enhanced CREB enrichment on the promoter, but AMPKα1 knockdown partially abrogated this binding (Figure 6J; Figure S15C). In summary, these results demonstrated that AMPKα1 activation delayed MSC aging and enhanced osteogenic commitment by promoting bone‐derived IGF‐1 secretion, and this process depended on CREB‐mediated transcriptional regulation (Figure 6K).

### 3.8 AMPKα1/CREB/IGF‐1 axis is essential to enhance osteogenic commitment of aged MSCs in 3D culture model

**FIGURE 7:**
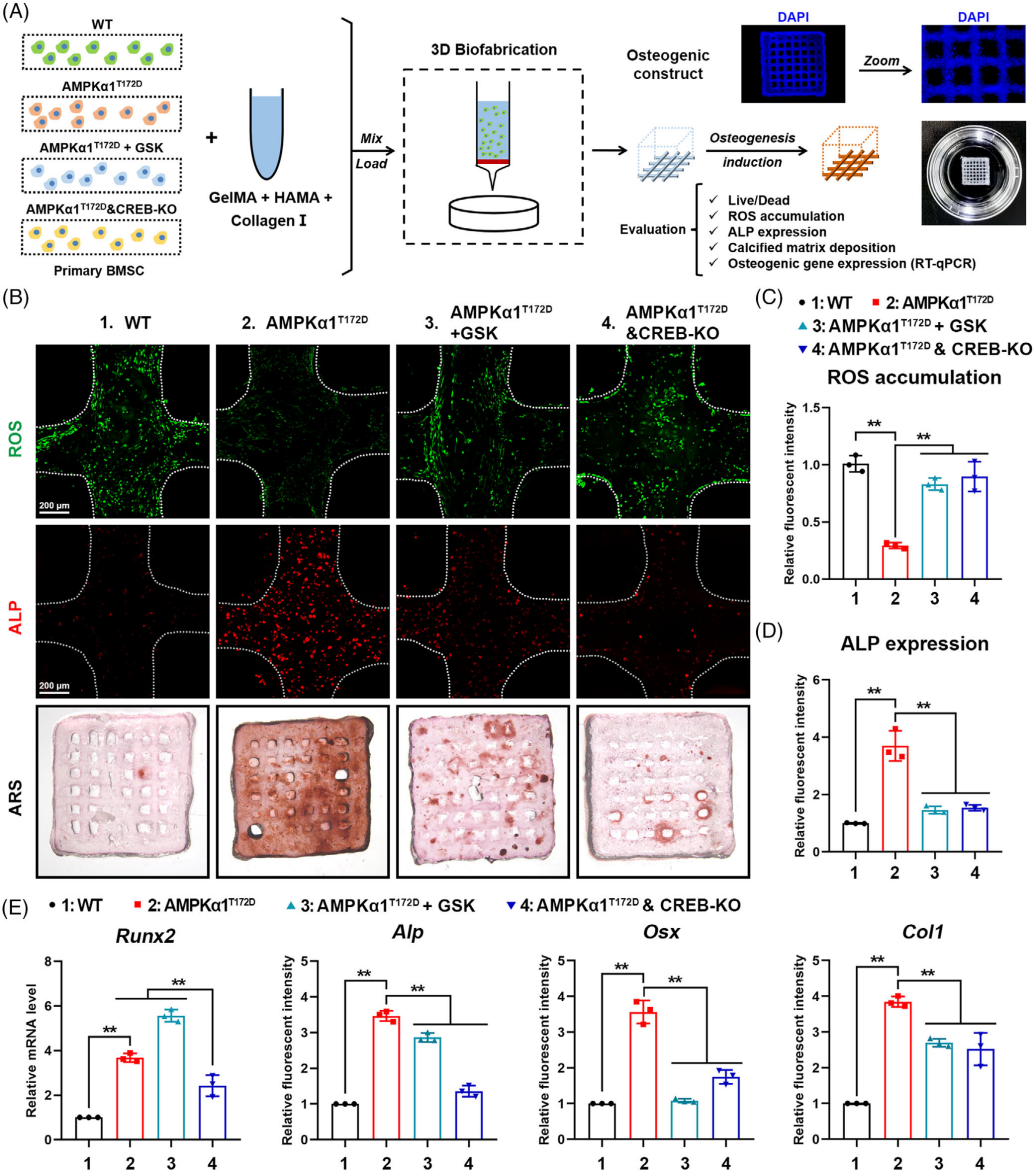
AMPKα1/CREB/IGF‐1 axis is essential to enhance osteogenic commitment of aged mesenchymal stem cells in 3D culture model. (A) Schematic illustration of study design for 3D bioprinting and for osteogenic induction and evaluation. (B) Representative images of intracellular reactive oxygen species (ROS) fluorescent staining, ALP immunofluorescent staining, and ARS staining of 3D culture models 21 days after printing. (C, D) Quantification of intracellular ROS level and ALP expression (*n* = 3). (E) Expression of osteogenic genes (*Runx2*, *Alp*, *Osx* and *Col1*) in 3D culture systems as detected by RT‐qPCR 21 days after printing (*n* = 3). 1: WT; 2: AMPKα1^T172D^; 3: AMPKα1^T172D^ + GSK; 4: AMPKα1^T172D^&CREB‐KO. Results are presented as bar plots with all data points. **p < 0.01.

In order to verify our findings from the 2D cell model again in the 3D culture environment, bioprinting technology was used to fabricate a 3D MSC culture model (Figure 7A). The characteristics of bone matrix bioink, the feasibility of bioprinting, and the evaluation workflow have been discussed in our previous studies.^15^ This 3D culture model offers a feasible ex vivo approach to investigating MSC osteogenic potential under various conditions. In the present study, four groups of primary MSCs were included: (1) aged WT MSCs; (2) aged AMPKα1^T172D^ MSCs; (3) aged AMPKα1^T172D^ MSCs treated with GSK; (4) aged AMPKα1^T172D^ MSCs with CREB‐KO by CRISPR‐Cas9. Of note, all groups of 3D culture models showed superior biocompatibility, as evidenced by >80% of cells remaining alive after 21 days' induction (Figure S16).

Consistent with results from the 2D culture system, constitutive AMPKα1 activation significantly attenuated ROS accumulation in 3D culture model compared with WT controls, but both GSK treatment and CREB‐KO increased ROS production to varying degrees (Figure 7B,C). ALP is a biomarker of osteogenic potential. We observed relatively more ALP^+^ MSCs in AMPKα1^T172D^ 3D culture models than in WT controls, whereas both GSK treatment and CREB‐KO diminished ALP expression (Figure 7B,D). Furthermore, we directly evaluated mineral deposition within 3D culture systems via ARS staining 21 days after induction. AMPKα1^T172D^ 3D culture models showed the most enriched calcium deposition, while both GSK treatment and CREB‐KO inhibited this deposition process (Figure 7B). At the transcriptional level, osteogenic‐marker expression (*Runx2*, *Alp*, *Osx* and *Col1*) in 3D models exhibited a similar trend: either CREB or IGF‐1 inactivation could impede the protective effect of constitutively activated AMPKα1 (Figure 7E). Collectively, these results once again confirmed the essential role of the AMPKα1/CREB/IGF‐1 axis in delaying MSC aging and promoting osteogenic commitment.

## 4 DISCUSSION

Bone formation requires energy consumption. Energy metabolism dysregulation is linked with bone loss and deterioration.^31^ Aging is accompanied by a synchronous decline in energy metabolism activity and bone formation capacity, but the relationship between energy metabolism and skeletal aging is still unclear.^32^ In this study, we provided first‐hand genetic evidence that the energy metabolism enzyme AMPK was involved in bone homeostasis during skeletal aging. Constitutive activation of AMPKα1 in *Prx1‐Cre* ^+^ MSCs led to increased bone‐derived IGF‐1 secretion and protected against age‐related bone loss, which we attributed to enhanced CREB‐mediated transcriptional regulation.

AMPK is the main cellular energy sensor and regulator. Previous studies have reported its vital role in bone remodelling, fracture healing, ectopic osteogenesis, and bone tissue engineering.^33^, ^34^, ^35^, ^36^ However, because of the prolonged time span required for aging research and the lack of long‐term AMPK activation strategy in vivo, few studies have focused on the protective effect of AMPK against skeletal aging.

Herein, we established an *Prx1* ^+^ MSC‐specific AMPK constitutive‐activation mouse model by introducing a phospho‐mimetic mutation (T172D) into the catalytic subunit AMPKα1. The activation effect of this mutation site has been confirmed by a wide range of studies.^37^, ^38^ At the animal level, we found that AMPKα1^T172D^ mice exhibited less age‐related bone loss, attenuated bone marrow adiposity, and increased numbers of viable osteocyte and periosteal osteoblast at the aged stage. At the cellular level, our data showed that T172D phospho‐mutation in aged MSCs enhanced cell self‐renewal and ALP^+^ colony formation capacity, decreased intracellular SA‐β‐gal and ROS accumulation, inhibited SASP, and further reactivated energy metabolism phenotype. Collectively, these data indicate that AMPKα1 activation modulates skeletal senescence.

It is intriguingly to notice that the size of ROS‐high MSCs seems larger than that of ROS‐low MSCs during in vitro culturing, and constitutive AMPK activation limited aged MSC enlargement. We speculate that cell size might positively correlate with the progression of MSCs senescence. Consistent with our findings, the work by Lengefeld et al. showed that stem cell enlargement led to reduced proliferation and impaired stemness during aging process^39^; the work by Wagner et al. reported that MSC replicative aging was accompanied by cell size increase, proliferation arrest and chromosomal instabilities.^40^ Therefore, cell size might be a determinant of MSC potential during aging, but the underlying mechanism still needs further research.

In addition to the skeleton, targeting AMPKα1 is also a feasible way to delay the aging processes of other organs. Rangarajan et al. reported that metformin reversed severe lung fibrosis in an AMPK‐dependent manner.^41^ Moreover, Zhang et al. found that AMPK activation delayed ovarian aging and improved female fertility.^42^ Together, these studies show that AMPKα1 is a potentially important therapeutic target for combating aging. As a next step, a novel agonist with better specificity and pharmacokinetic features should be developed.

Notably, no significant differences in bone volume, bone microarchitecture, bone marrow adiposity, or number of osteogenic cells was observed between AMPKα1^T172D^ mice and WT controls at 3‐month‐old age. These results implied that congenital AMPKα1 activation may not directly affect mouse skeletal development before adulthood. Therefore, AMPKα1 activation‐mediated protective effects on 22‐month‐old mouse skeletons could be mainly attributed to delaying skeletal aging. The study by Ruiz et al. reported that AMPK activity peaks early, is sustained during young adulthood, and declines gradually with aging.^43^ Considering the kinase cascade characteristic of feedback inhibition, we speculate that phospho‐mimetic mutation increased the reserve capacity of AMPK cascade to function until the moment that intrinsic AMPK activity is diminished in old age. Our hypothesis is supported by a previous study that found that AMPKα1 ablation during the embryonic period did not affect mouse endochondral ossification or bone development.^44^ Thus, the beneficial effect of AMPK activation is strongly age related.

IGF‐1 is the most common growth factor deposited in bone throughout a mammal's lifetime. Its secretion declines with time; very low levels are detected in older adults. This phenomenon is called somatopause.^26^ Intriguingly, IGF‐1 plays dual roles in regulating cell senescence. Although overdose of IGF‐1 leads to inflammatory reaction and metabolic disorder, an appropriate amount thereof is essential for scavenging intracellular ROS and maintaining cellular mitochondrial biogenesis.^45^ In the skeletal system, bone‐derived IGF‐1 contributes to bone homeostasis in an autocrine/paracrine manner. Xian et al. reported that matrix IGF‐1 enhanced osteogenic commitment and maintained bone mass.^12^ This result was once again confirmed in the current study.

In the past few years, many studies have investigated the downstream signalling mechanisms of bone‐derived IGF‐1, but few have focused on the upstream regulator of bone‐derived IGF‐1 expression. CREB is a well‐known transcription factor linked to cellular growth and development.^46^ In several cell types, CREB has been documented as involved in transcriptional regulation of crucial genes such as insulin, growth factor, or cyclin.^47^, ^48^ Notably, Thomson et al. first identified CREB as a direct downstream target of AMPK.^30^ Activated AMPK phosphorylates CREB at Ser133 and promotes its nuclear translocation. Interestingly, previous papers have shown that CREB also participates in IGF‐1 downstream signalling.^49^ Therefore, CREB is not only an activator of IGF‐1 expression but also a downstream effector of IGF‐1 signalling. This forms a positive feedback loop that augments AMPK function.

There are still some limitations and drawbacks in the present study. First, due to the limitation of construction strategy, the transgenic mice express mutated AMPKα1 from the birth. Even though no significant differences in skeletal phenotype were observed between 3‐month‐old transgenic mice and WT controls, it was still difficult to distinguish indirect contributions of congenital AMPKα1 activation between development and aging. Hopefully, this problem may be overcome through the application of a *CreERT*‐mediated inducible knockout strategy, in which *Cre* is fused with a functional oestrogen receptor, to be activated by tamoxifen only in the aging process. Second, although data prove our findings in a cell model, 3D culture system, and transgenic mouse model, evidence from human study is still lacking. As a next step, we plan to develop specific, long‐term AMPKα1 agonists for clinical translational research.

In conclusion, the findings of this study highlighted AMPKα1 as a critical regulatory protein in protection against skeletal aging. We found that constitutive AMPKα1 activation delaying murine MSC aging and enhanced their osteogenic commitment by promoting CREB‐mediated IGF‐1 secretion. Further studies are still needed to validate whether AMPKα1 could be a direct therapeutic target for the clinical SOP treatment.

## AUTHOR CONTRIBUTIONS

Yiqi Yang and Kai Yuan contributed equally to this work. *Conceptualization*: Yiqi Yang, Yugang Wang, Qiming Fan, Hanjun Li and Tingting Tang; *Study conduct*: Yiqi Yang, Kai Yuan, Yihao Liu, Qishan Wang, Yixuan Lin, Shengbing Yang, Kai Huang, Tianyou Kan, Yuxin Zhang and Mingming Xu; *Data analysis*: Yiqi Yang, Kai Yuan, Qishan Wang and Zhifeng Yu; *Writing*: Yiqi Yang and Kai Yuan; *Supervision*: Yugang Wang, Hanjun Li and Tingting Tang. All authors reviewed and approved the final manuscript.

## FUNDING INFORMATION

This study was financially supported by the Foundation for Innovative Research Groups of the National Science Foundation of China (grants 81921002), the National Natural Science Foundation of China (grants 92068205 and 82002328) and the China Postdoctoral Science Foundation (grants 2018M632136 and 2019T120348). We thank Shanghai Graphic Design Information Co., Ltd. (Shanghai, China) for providing 3D bioprinting technical support.

## CONFLICT OF INTEREST STATEMENT

The authors have no conflicts of interest to declare.

## Supporting information


**Figure S1.** Schematic illustration of the activation relationship between AMPKα1 and ACC.
**Figure S2.** Surface marker expression of primary MSCs at passage 6 by flow cytometric analysis.
**Figure S3.** Quantification of WB shown in Figure 1.
**Figure S4.** MSC lineage commitment is accompanied by changes in AMPKα1 activity.
**Figure S5.** Analysis of cortical bone and osteoclastogenesis in vivo.
**Figure S6.** Quantification of metabolic parameters in Mito stress test and Glycolysis stress test.
**Figure S7.** Visualization of differentially expressed genes in sequencing.
**Figure S8.** AMPKα1 controls *Igf1* mRNA expression in primary MSCs and C3H10T1/2 cells.
**Figure S9.** Validation of AMPKα1 constitutive activation and AMPKα1 knockdown.
**Figure S10.** Quantification of WB shown in Figure 4.
**Figure S11.** Osteogenic and adipogenic gene expression in MSCs with various treatments.
**Figure S12.** Effects of exogenous IGF‐1 on AMPKα1‐knockdown C3H10T1/2 cell differentiation.
**Figure S13.** Quantification of WB shown in Figure 6.
**Figure S14.** Validation of CREB overexpression and knockout.
**Figure S15.** CREB is essential for IGF‐1 expression in shAMPKα1‐2 cell line.
**Figure S16.** Cell survival in 3D‐bioprinting osteogenic construct.
**Table S1.** Primer sequences used in RT‐qPCR.
**Table S2.** Primer sequences used in cell line construction.
**Table S3.** Primer sequences used in ChIP assay.

## Data Availability

The data that support the findings of this study are available from the corresponding author upon reasonable request.
