# Supplementary material for: Constitutively activated AMPKα1 protects against skeletal aging in mice by promoting bone‐derived IGF‐1 secretion
Source: Cell Prolif. 2023 Apr 11;56(10):e13476. doi: 10.1111/cpr.13476 (PMC10542616; doi:10.1111/cpr.13476)
Supplement: Supplementary file 1 — Figure S1. Schematic illustration of the activation relationship between AMPKα1 and ACC. Figure S2. Surface marker expression of primary MSCs at passage 6 by flow cytometric analysis. Figure S3. Quantification of WB shown in Figure 1. Figure S4. MSC lineage commitment is accompanied by changes in AMPKα1 activity. Figure S5. Analysis of cortical bone and osteoclastogenesis in vivo. Figure S6. Quantification of metabolic parameters in Mito stress test and Glycolysis stress test. Figure S7. Visualization of differentially expressed genes in sequencing. Figure S8. AMPKα1 controls Igf1 mRNA expression in primary MSCs and C3H10T1/2 cells. Figure S9. Validation of AMPKα1 constitutive activation and AMPKα1 knockdown. Figure S10. Quantification of WB shown in Figure 4. Figure S11. Osteogenic and adipogenic gene expression in MSCs with various treatments. Figure S12. Effects of exogenous IGF‐1 on AMPKα1‐knockdown C3H10T1/2 cell differentiation. Figure S13. Quantification of WB shown in Figure 6. Figure S14. Validation of CREB overexpression and knockout. Figure S15. CREB is essential for IGF‐1 expression in shAMPKα1‐2 cell line. Figure S16. Cell survival in 3D‐bioprinting osteogenic construct. Table S1. Primer sequences used in RT‐qPCR. Table S2. Primer sequences used in cell line construction. Table S3. Primer sequences used in ChIP assay. [file CPR-56-e13476-s001.docx]

Supporting Information

**Constitutively Activated AMPKα1 Protects Against Skeletal Aging in Mice by Promoting Bone-derived IGF-1 Secretion**

*Yiqi Yang^#^, Kai Yuan^#^, Yihao Liu, Qishan Wang, Yixuan Lin, Shengbing Yang, Kai Huang, Tianyou Kan, Yuxin Zhang, Mingming Xu, Zhifeng Yu, Qiming Fan, Yugang Wang^*^, Hanjun Li^*^* and *Tingting Tang^*^*

**Fig. S1.** Schematic illustration of the activation relationship between AMPKα1 and ACC

**Fig. S2.** Surface marker expression of primary MSCs at passage 6 by flow cytometric analysis

**Fig. S3.** Quantification of WB shown in Figure 1

**Fig. S4.** MSC lineage commitment is accompanied by changes in AMPKα1 activity

**Fig. S5.** Analysis of cortical bone and osteoclastogenesis *in vivo*

**Fig. S6.** Quantification of metabolic parameters in Mito stress test and Glycolysis stress test

**Fig. S7.** Visualization of differentially expressed genes in sequencing

**Fig. S8.** AMPKα1 controls *Igf1* mRNA expression in primary MSCs and C3H10T1/2 cells

**Fig. S9.** Validation of AMPKα1 constitutive activation and AMPKα1 knockdown

**Fig. S10.** Quantification of WB shown in Figure 4

**Fig. S11.** Osteogenic and adipogenic gene expression in MSCs with various treatments

**Fig. S12.** Effects of exogenous IGF-1 on AMPKα1-knockdown C3H10T1/2 cell differentiation

**Fig. S13.** Quantification of WB shown in Figure 6

**Fig. S14.** Validation of CREB overexpression and knockout

**Fig. S15.** CREB is essential for IGF-1 expression in shAMPKα1-2 cell line

**Fig. S16.** Cell survival in 3D-bioprinting osteogenic construct

**Table S1.** Primer sequences used in RT-qPCR

**Table S2.** Primer sequences used in cell line construction

**Table S3.** Primer sequences used in ChIP assay


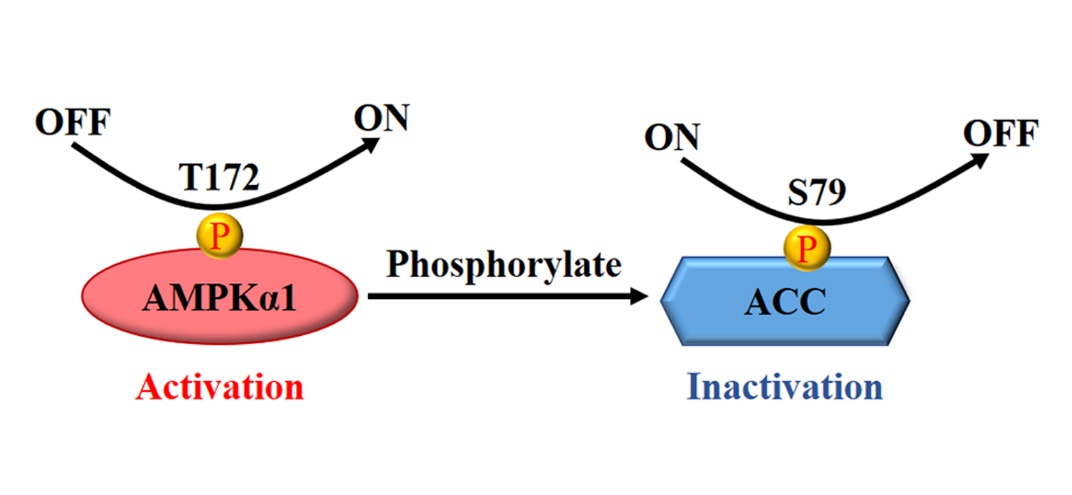


**Fig. S1.** Schematic illustration of the activation relationship between AMPKα1 and ACC. AMPKα1-T172 phosphorylation (pAMPKα1-T172) indicates AMPKα1 activation, and then activated AMPK directly phosphorylates ACC at S79 (pACC-S79). Thus, pACC-S79 expression level is proportional to its upstream AMPKα1 activity.

**
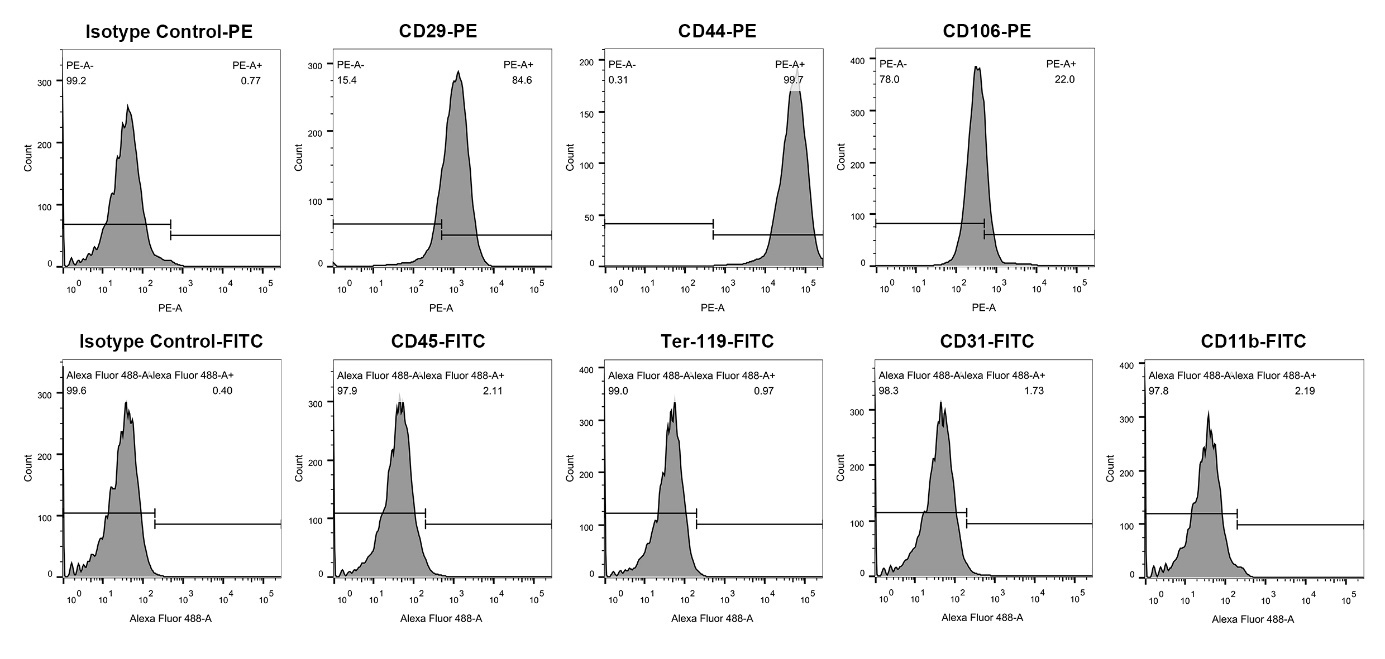
**

**Fig. S2.** Surface marker expression of primary MSCs at passage 6 by flow cytometric analysis. MSCs were isolated from the bone marrow of mice and serially passaged using MesenCult Expansion Kit *in vitro*. At passage 6, MSCs were collected and probed for MSC-specific biomarker expression. Primary MSCs at passage 6 demonstrated a high expression of CD29 (84.6%), CD44 (99.7%) and CD106 (22.0%), but they express CD45 (2.11%), Ter-119 (0.97%), CD31 (1.73%) and CD11b (2.19%) negatively. This indicated that MSCs maintained their stem cell phenotype during serial passaging *in vitro*.

**
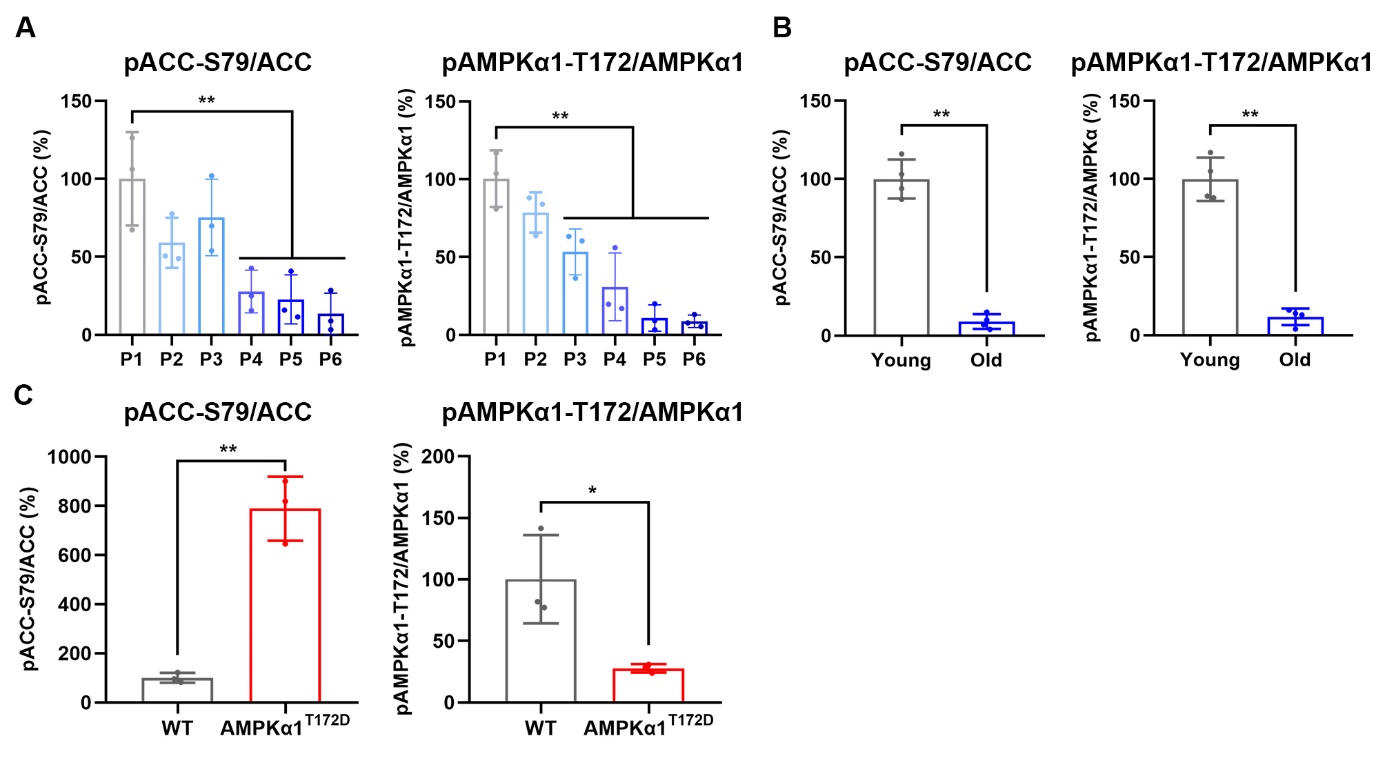
**

**Fig. S3.** Quantification of WB shown in Figure 1. (A) Quantitative analysis of WB bands from Figure 1A. (B) Quantitative analysis of WB bands from Figure 1B. (C) Quantitative analysis of WB bands from Figure 1H. Results are presented as bar plots with all data points. ^*^*p* < 0.05; ^**^*p* < 0.01.

**
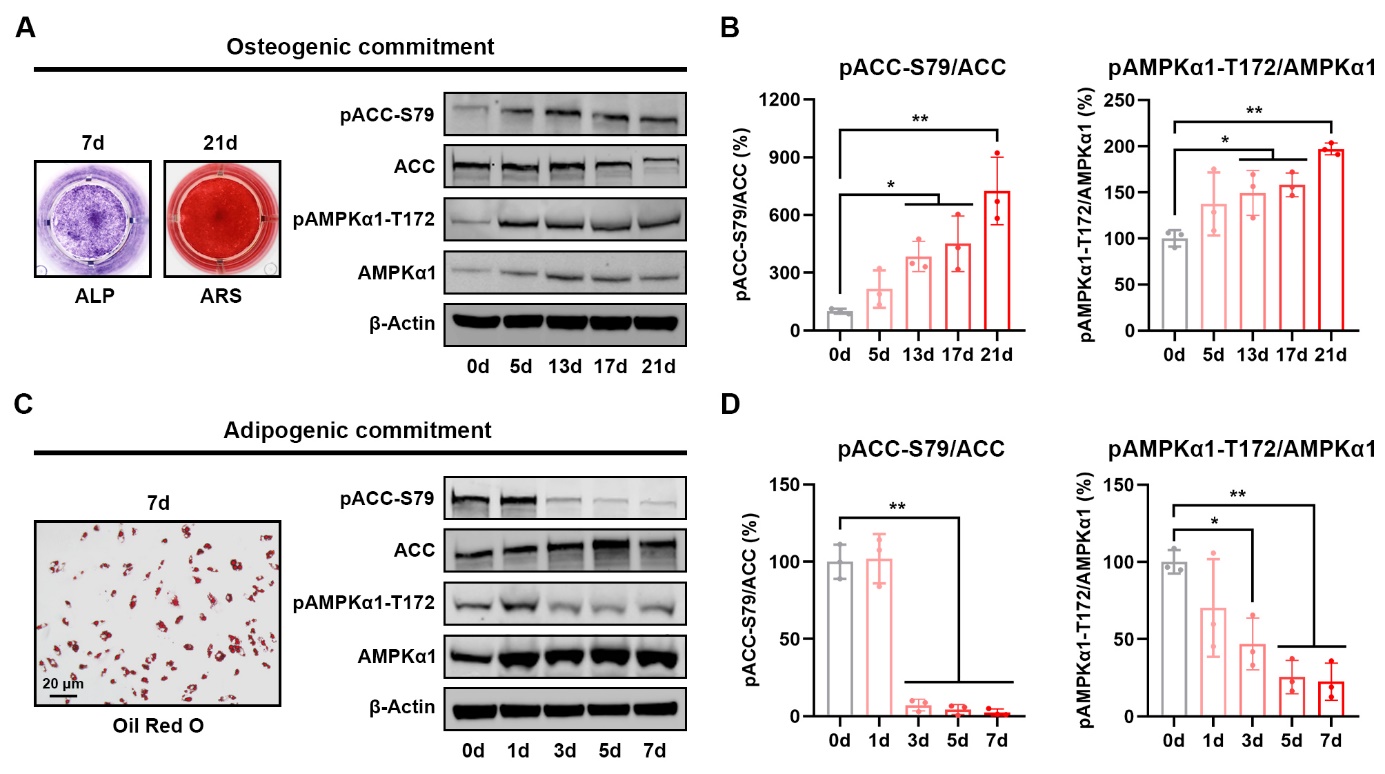
**

**Fig. S4.** MSC lineage commitment is accompanied by changes in AMPKα1 activity. (A) Representative images of ALP staining at day 7 and ARS staining at day 21 after osteogenic induction. Immunoblotting showing increased expression of pAMPKα1-T172 and pACC-S79 during osteogenic commitment. Samples were collected at 0, 5, 13, 17, and 21 days. (B) Quantitative analysis of WB bands shown in (A). (C) Representative images of Oil Red O staining at day 7 after adipogenic induction. Immunoblotting showing decreased expression of pAMPKα1-T172 and pACC-S79 during adipogenic commitment. Samples were collected at 0, 1, 3, 5, and 7 days. (D) Quantitative analysis of WB bands shown in (C). Results are presented as bar plots with all data points. ^*^*p* < 0.05; ^**^*p* < 0.01.

**
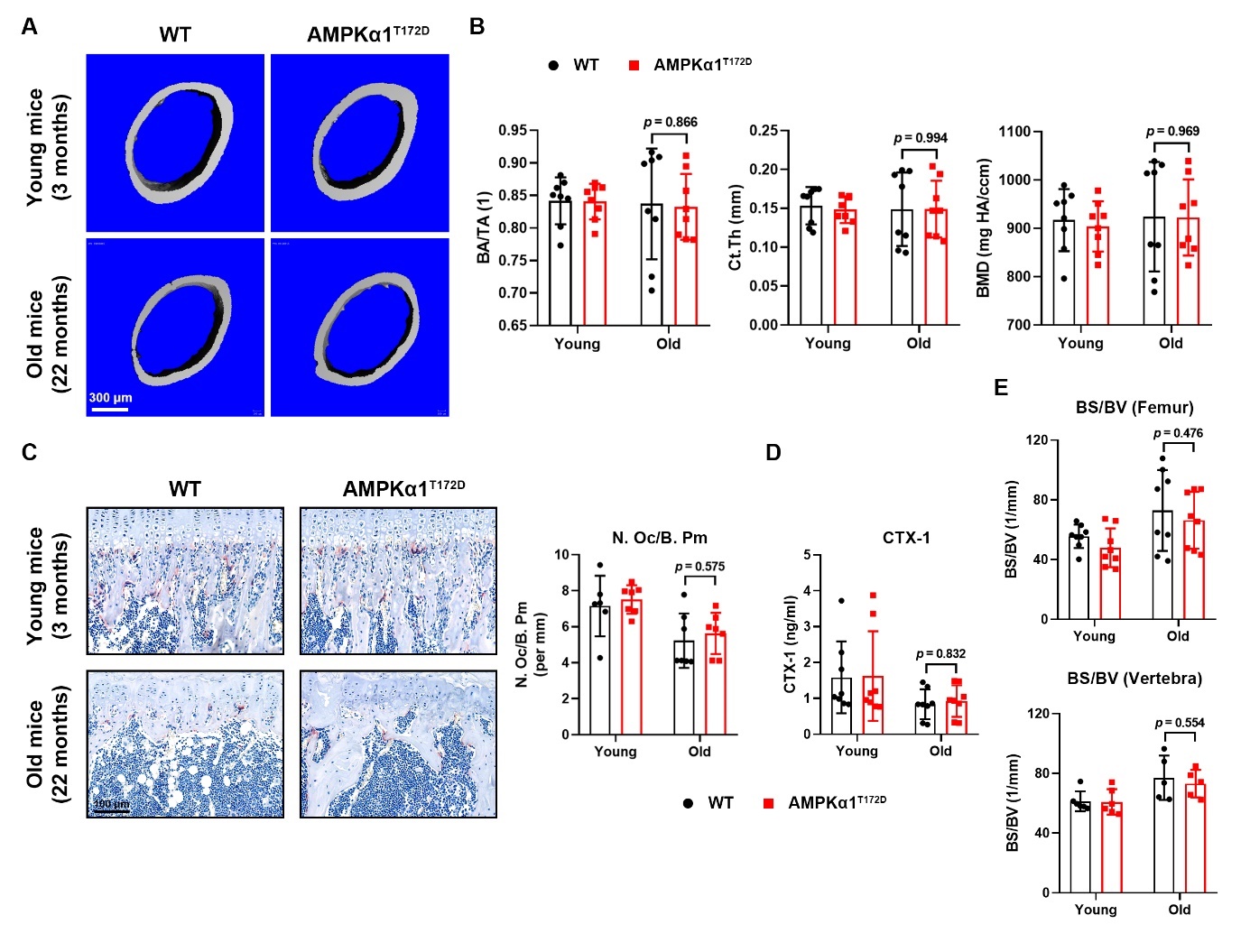
**

**Fig. S5.** Analysis of cortical bone and osteoclastogenesis *in vivo*. (A) 3D μCT images of femur midshafts from AMPKα1^T172D^ mice and WT controls. (B) Quantitative μCT morphometric analysis of cortical bones (n = 8). (C) TRAP staining images and quantitative analyses of TRAP^+^ cells on trabecular bone (n = 6-7). (D) Serum CTX-1 concentrations of AMPKα1^T172D^ mice and WT littermate controls (n = 8). (E) Quantitative analysis of bone surface / bone volume (BS/BV) of femur trabecular bones (n = 8) and vertbral bodies (n = 5-6) based on μCT. Results are presented as bar plots with all data points.


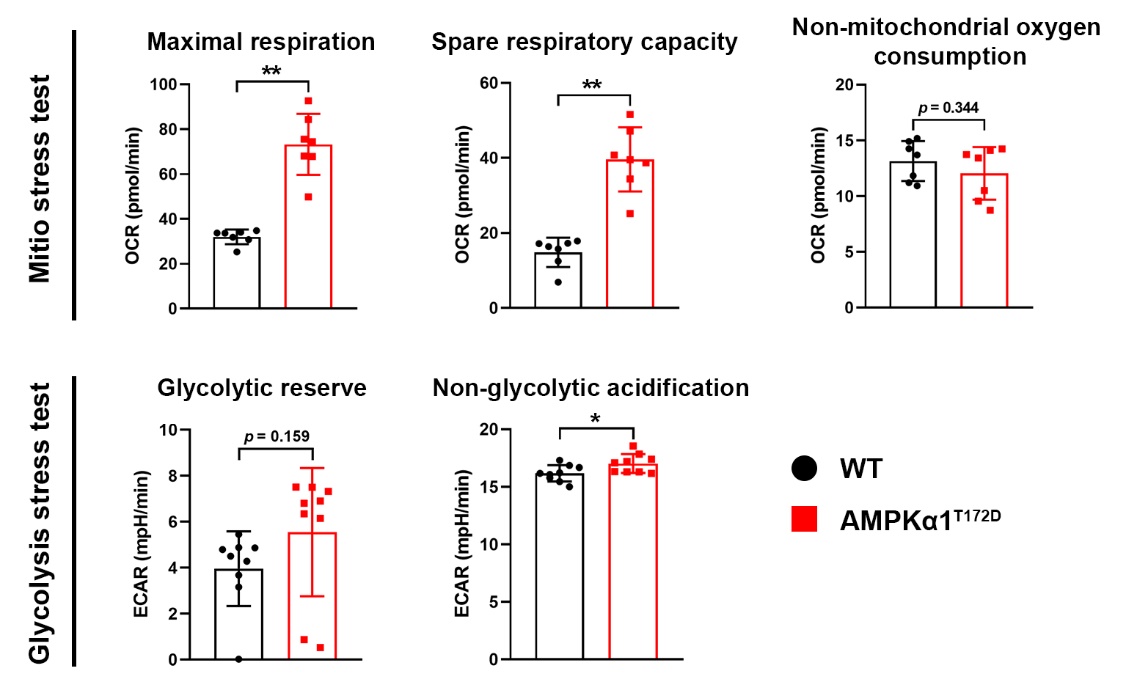


**Fig. S6.** Quantification of metabolic parameters in Mito stress test and Glycolysis stress test. Top: Quantitative analysis of maximal respiration, spare respiratory capacity and non-mitochondrial oxygen consumption based on OCR (n = 7). Bottom: Quantitative analysis of glycolytic reverse and non-glycolytic acidification based on ECAR (n = 9). Results are presented as bar plots with all data points. ^*^*p* < 0.05; ^**^*p* < 0.01.


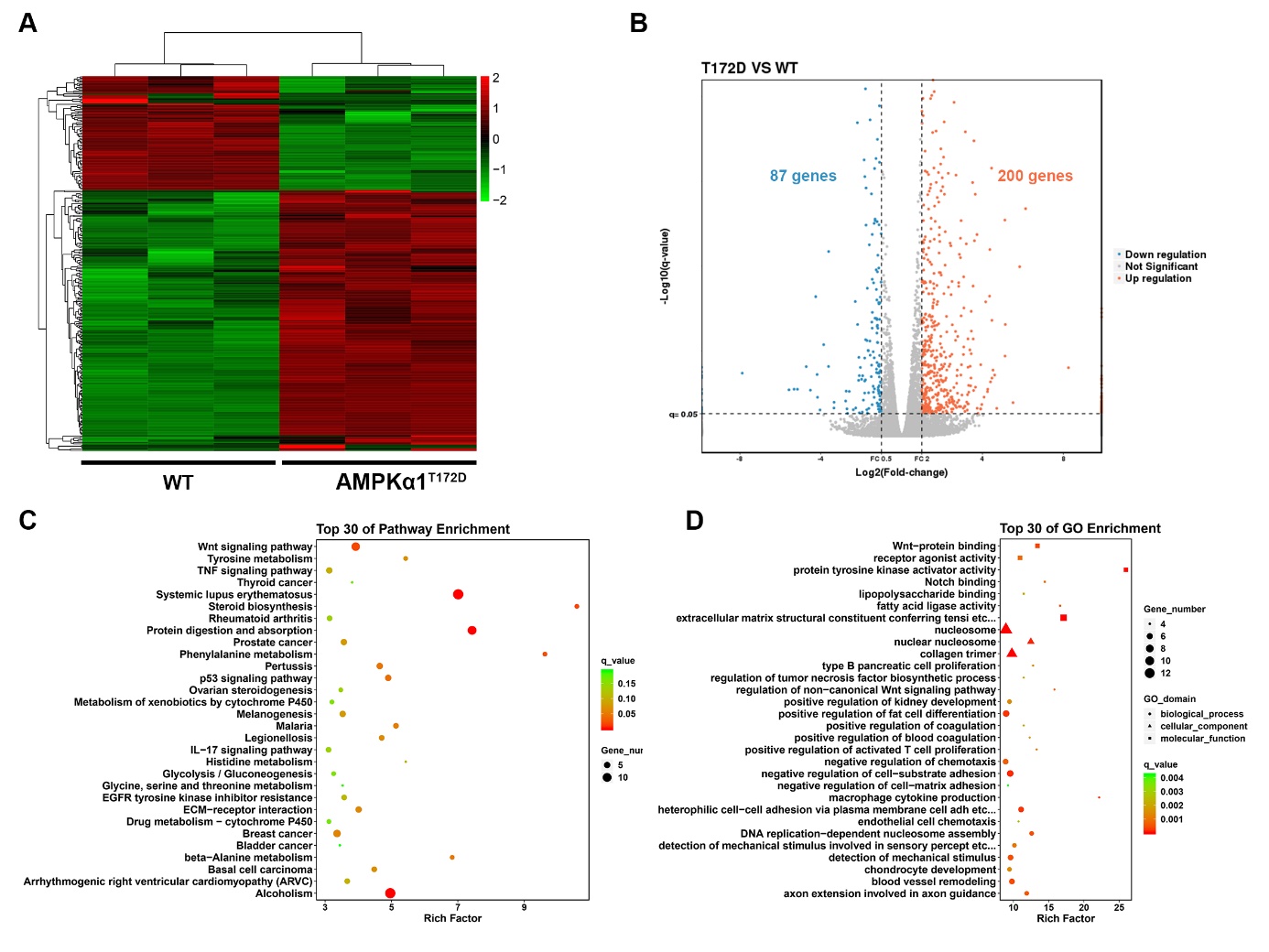


**Fig. S7.** Visualization of differentially expressed genes in sequencing. (A) Heatmap showing differentially expressed genes between AMPKα1^T172D^ mice and WT controls. (B) Volcano map showing differentially expressed genes between AMPKα1T172D mice and WT controls. (C) Top 30 KEGG pathway enrichments of differentially expressed genes. (D) Top 30 GO enrichments of differentially expressed genes.


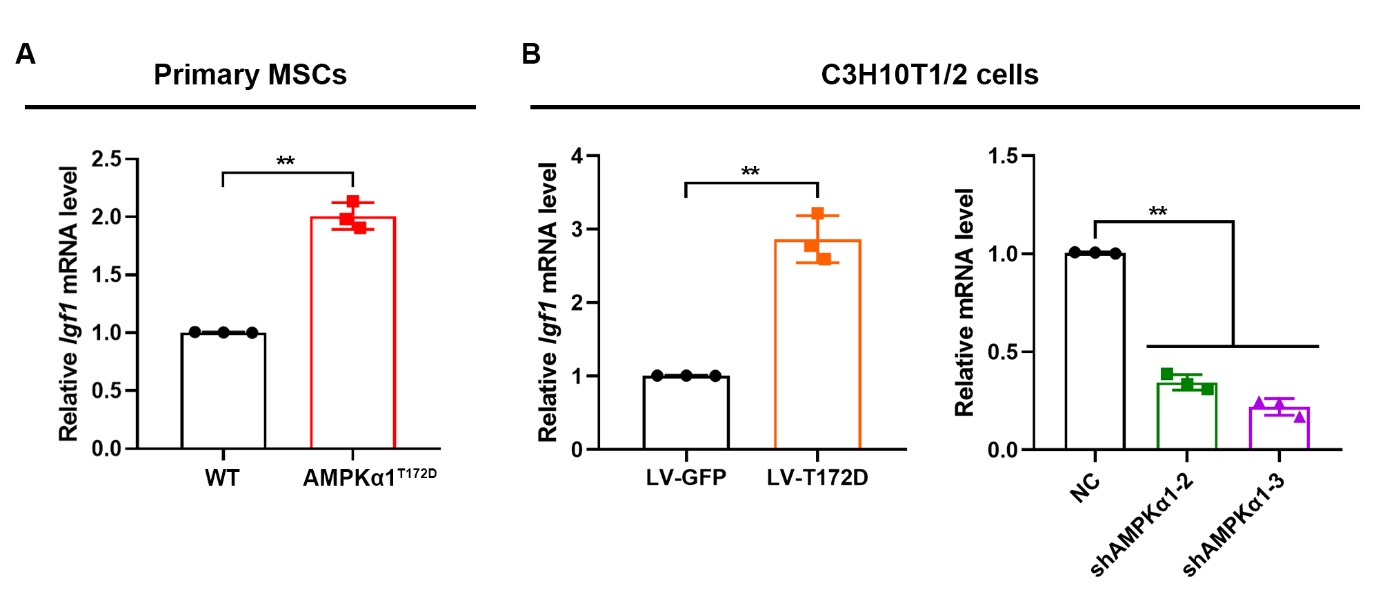


**Fig. S8.** AMPKα1 controls *Igf1* mRNA expression in primary MSCs and C3H10T1/2 cells. (A) Effects of AMPKα1 constitutive activation on *Igf1* mRNA expression in primary MSCs as detected by RT-qPCR (n =3). (B) Effects of AMPKα1 constitutive activation or AMPKα1 knockdown on *Igf1* mRNA expression in C3H10T1/2 cells as detected by RT-qPCR (n =3). Results are presented as bar plots with all data points. ^**^*p* < 0.01.


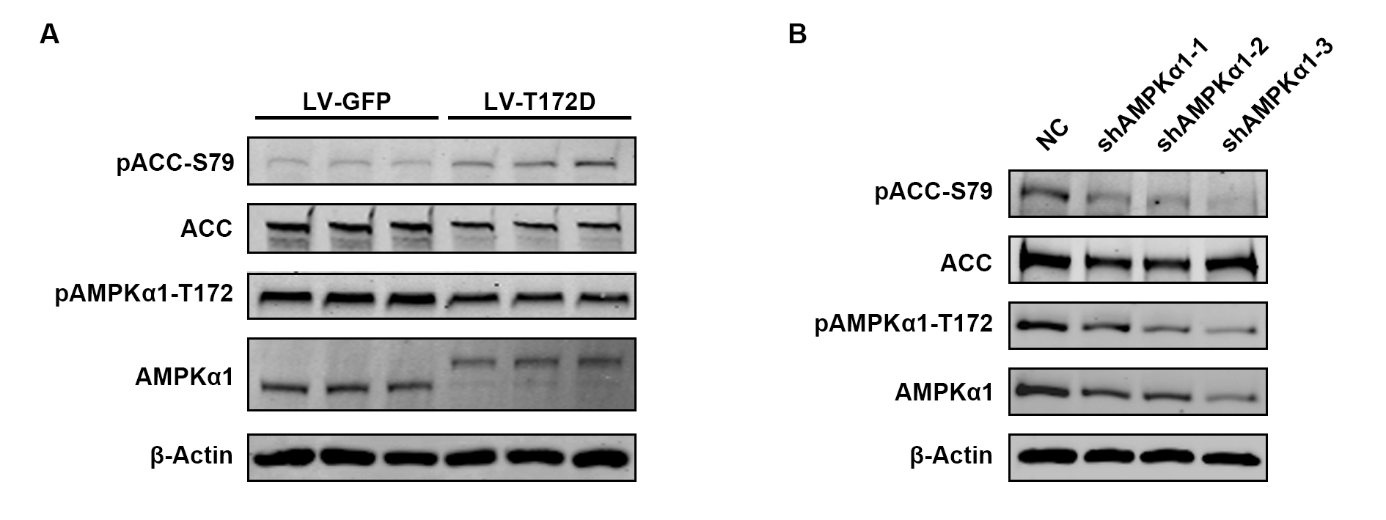


**Fig. S9.** Validation of AMPKα1 constitutive activation and AMPKα1 knockdown. (A) The expression of pACC-S79, ACC, pAMPKα1-T172, and AMPKα1 after AMPKα1 constitutive activation in C3H10T1/2 cells as detected by immunoblotting. (B) The expression of pACC-S79, ACC, pAMPKα1-T172, and AMPKα1 after AMPKα1 knockdown in C3H10T1/2 cells as detected by immunoblotting.


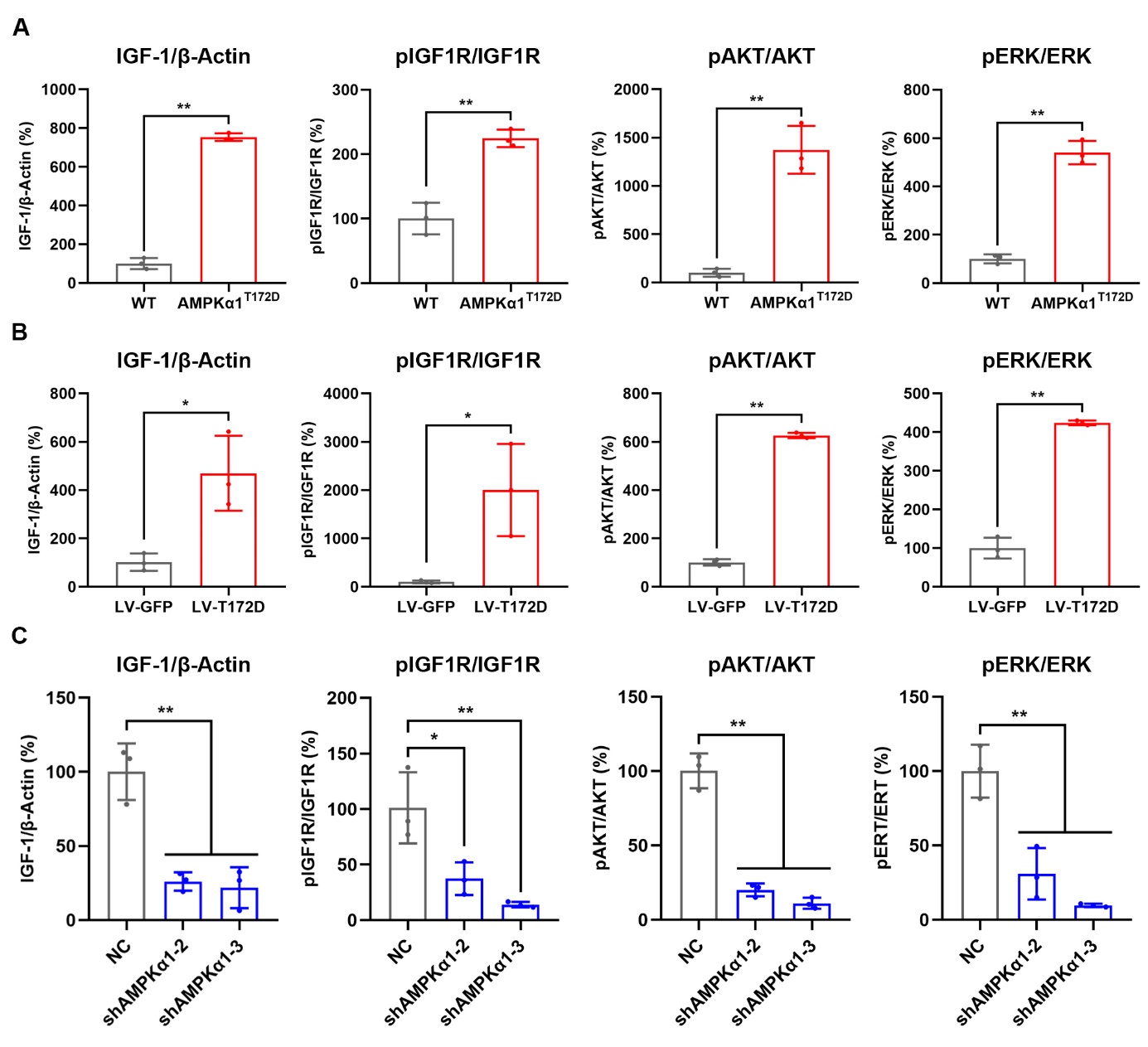


**Fig. S10.** Quantification of WB shown in Figure 4. (A) Quantitative analysis of WB bands from Figure 4E. (B) Quantitative analysis of WB bands from Figure 4F. (C) Quantitative analysis of WB bands from Figure 4G. Results are presented as bar plots with all data points. ^*^*p* < 0.05; ^**^*p* < 0.01.


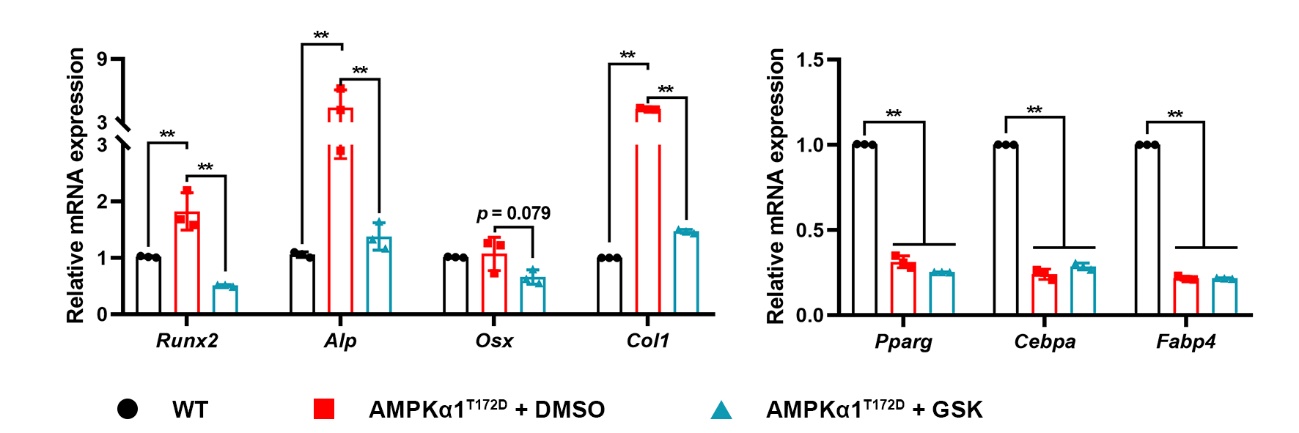


**Fig. S11.** Effects of GSK on expression of osteogenic (*Runx2*, *Alp*, *Osx*, and *Col1*) and adipogenic (*Pparg*, *Cebpa*, and *Fabp4*) markers as assessed by RT-qPCR in MSCs with constitutive AMPKα1 activation (n = 3). Results are presented as bar plots with all data points. ^**^*p* < 0.01.


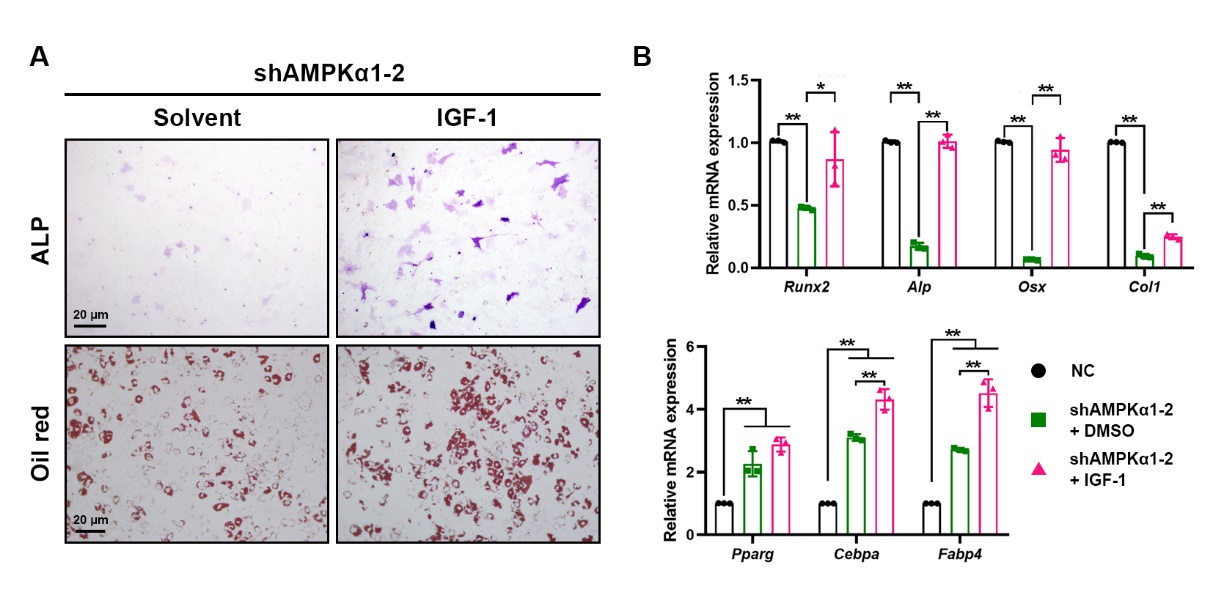


**Fig. S12.** Effects of exogenous IGF-1 on AMPKα1-knockdown C3H10T1/2 cell differentiation. (A) AMPKα1 was knockdown in C3H10T1/2 cells (shAMPKα1-2). Cell differentiation was assessed by oil red staining at day 7 during adipogenic induction or by ALP staining at day 14 during osteogenic induction. (B) Effects of exogenous IGF-1 on the expression of osteogenic (*Runx2*, *Alp*, *Osx* and *Col1*) and adipogenic (*Pparg*, *Cebpa*, and *Fabp4*) markers as assessed by RT-qPCR in C3H10T1/2 cells with AMPKα1 knockdown (n = 3). Results are presented as bar plots with all data points. ^*^*p* < 0.05; ^**^*p* < 0.01.


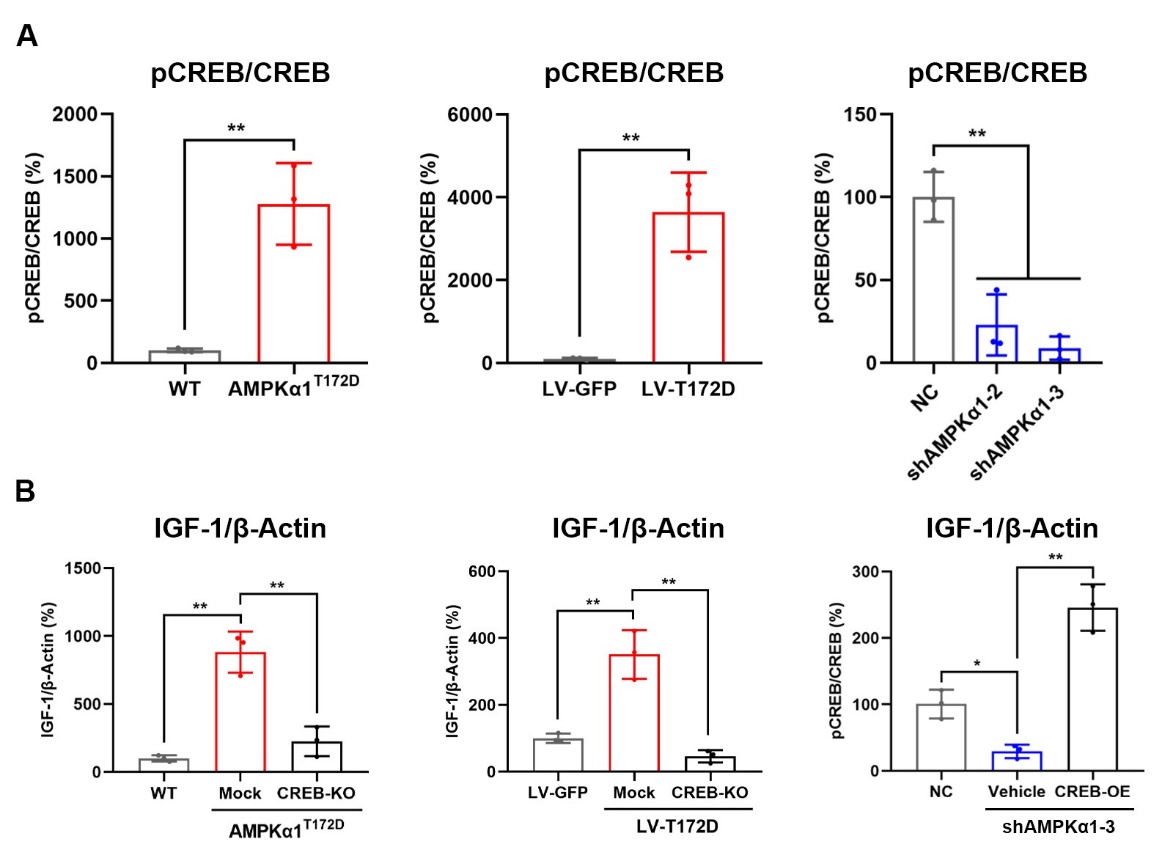


**Fig. S13.** Quantification of WB shown in Figure 6. (A) Quantitative analysis of WB bands from Figure 6A-6C. (B) Quantitative analysis of WB bands from Figure 6D. Results are presented as bar plots with all data points. ^*^*p* < 0.05; ^**^*p* < 0.01.


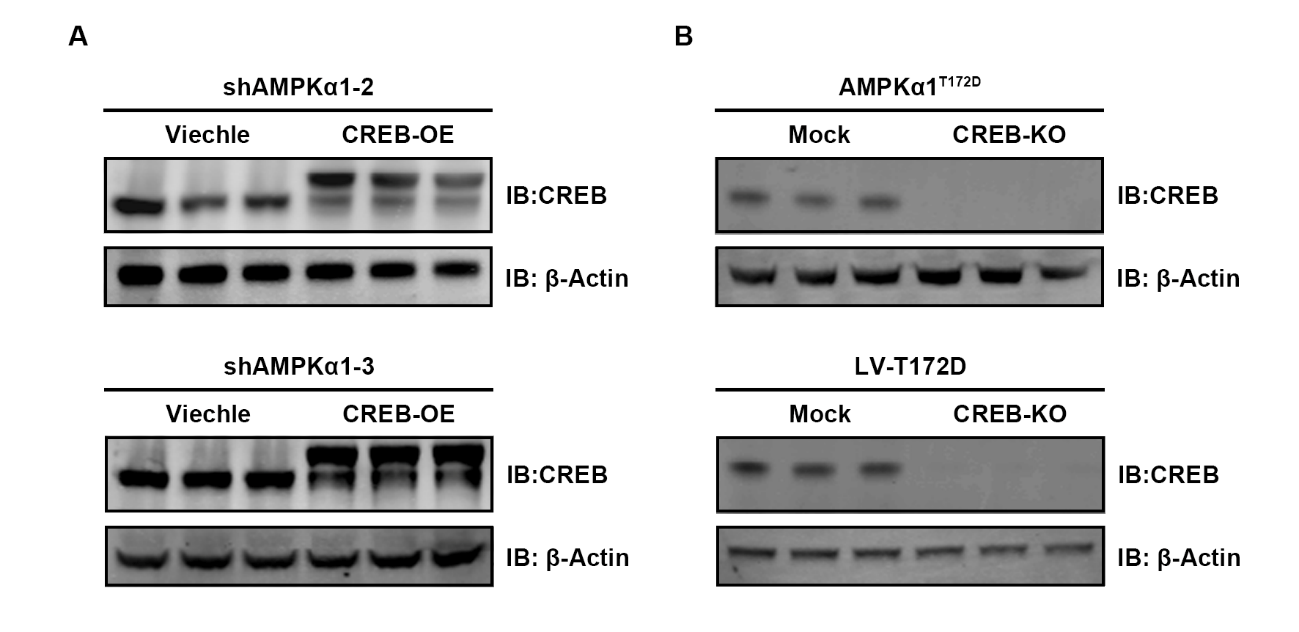


**Fig. S14.** Validation of CREB overexpression and knockout. (A) The expression of CREB after CREB overexpression in C3H10T1/2 cells as detected by immunoblotting. (B) The expression of CREB after CREB knockout in MSCs and C3H10T1/2 cells as detected by immunoblotting.


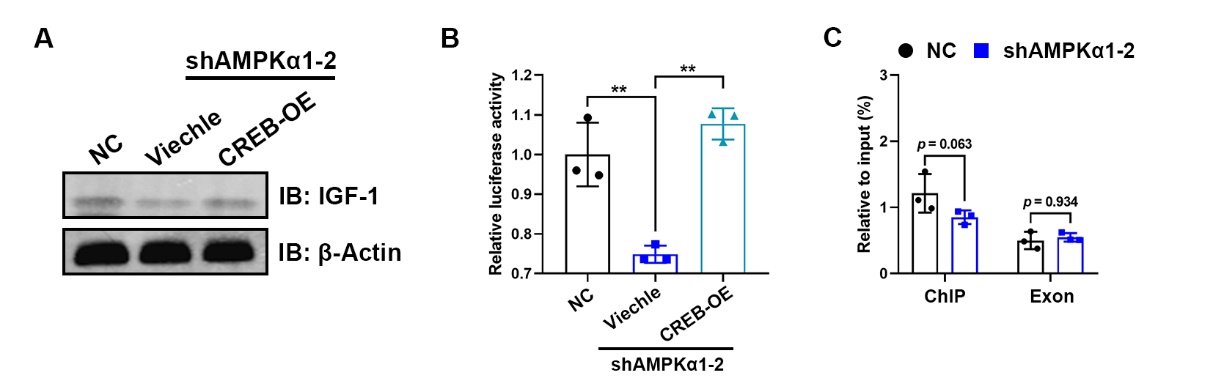


**Fig. S15.** CREB is essential for IGF-1 expression in shAMPKα1-2 cell line. (A) Effects of CREB overexpression on IGF-1 protein expression in shAMPKα1-2 cells as detected by immunoblotting. (B) Effects of CREB overexpression on *Igf1* promoter transcriptional activity in shAMPKα1-2 cells as detected by dual-luciferase reporter assays (n = 3). (C) ChIP analysis of CREB binding to the *Igf1* promoter (-837 to -826) and exon region (n=3). Results are presented as bar plots with all data points. ^**^*p* < 0.01.


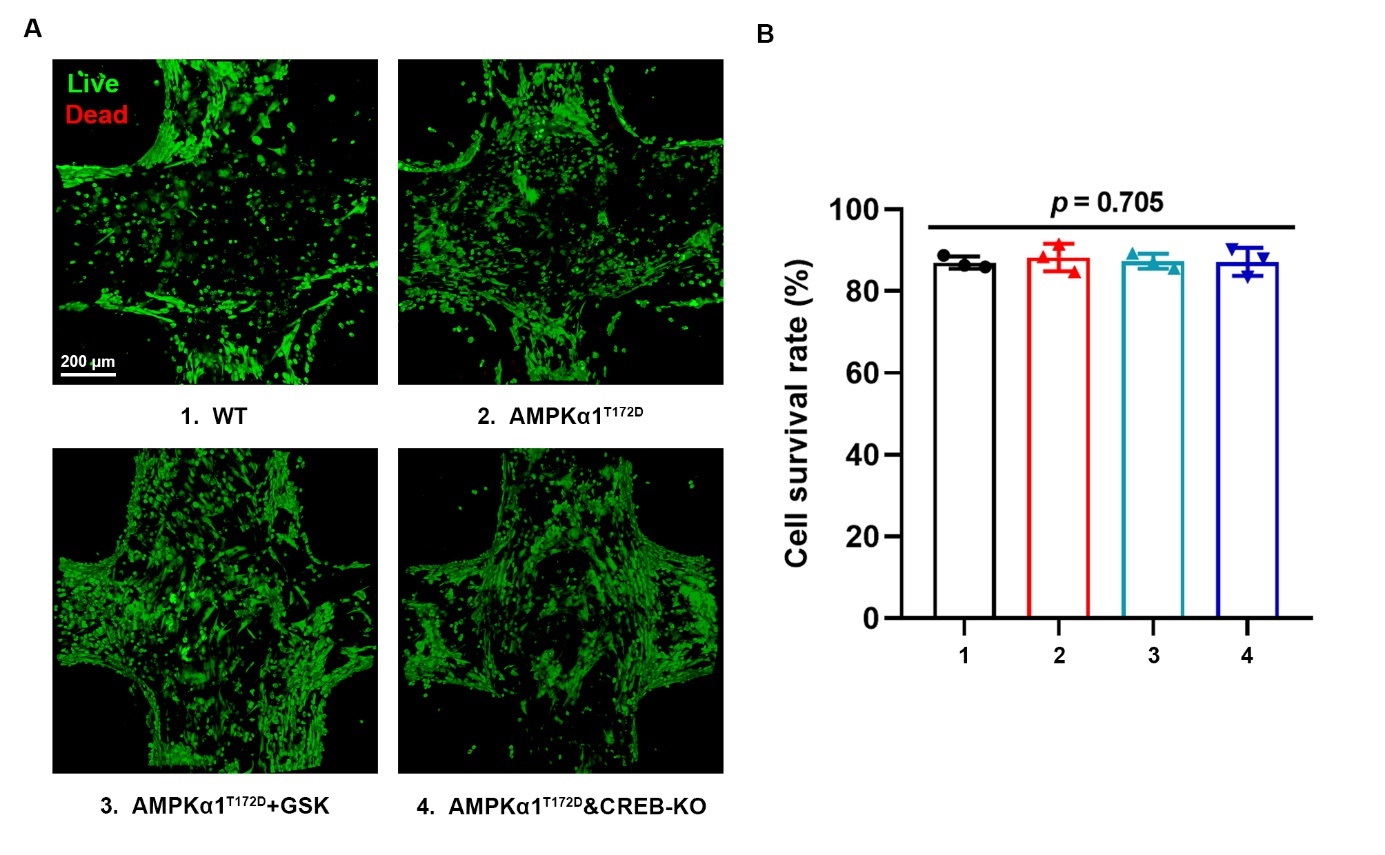


**Fig. S16.** Cell survival in 3D culture systems. (A) Representative fluorescent images of MSC Live/Dead staining under various conditions 21 days after printing. (B) Quantification of cell survival rate (n = 3). 1: WT; 2: AMPKα1^T172D^; 3: AMPKα1^T172D^ + GSK; 4: AMPKα1^T172D^&CREB-KO. Results are presented as bar plots with all data points.

**Table S1.** Primer sequences used in RT-qPCR.

| Gene | Sequence | |
| --- | --- | --- |
| β-Actin | Forward | AGAGGGAAATCGTGCGTGACA |
|  | Reverse | CACTGTGTTGGCATAGAGGTC |
| Pparg | Forward | GGAAAGACAACGGACAAATCAC |
|  | Reverse | TACGGATCGAAACTGGCAC |
| Cebpa | Forward | TGGACAAGAACAGCAACGAG |
|  | Reverse | TCACTGGTCAACTCCAGCAC |
| Fabp4 | Forward | GATGAAATCACCGCAGACGACA |
|  | Reverse | ATTGTGGTCGACTTTCCATCCC |
| Alp | Forward | GCCTGGATCTCATCAGTATTTGG |
|  | Reverse | GTTCAGTGCGGTTCCAGACAT |
| Col1 | Forward | CCGGAAGAATACGTATCACC |
|  | Reverse | ACCAGGAGGACCAGGAAGTC |
| Runx2 | Forward | CCGGGAATGATGAGAACTA |
|  | Reverse | ACCGTCCACTGTCACTTT |
| Osterix | Forward | CTCTCTGCTTGAGGAAGAAG |
|  | Reverse | GTCCATTGGTGCTTGAGAAG |
| Igf1 | Forward | CTGGACCAGAGACCCTTTGC |
|  | Reverse | GGACGGGGACTTCTGAGTCTT |
| p16 | Forward | CGCAGGTTCTTGGTCACTGT |
|  | Reverse | TGTTCACGAAAGCCAGAGCG |
| p21 | Forward | CAGACCAGCCTGACAGAT |
|  | Reverse | TGACCCACAGCAGAAGAG |
| Tnfa | Forward | CCCTCACACTCAGATCATCTTCT |
|  | Reverse | GCTACGACGTGGGCTACAG |
| Il-8 | Forward | CAAGGCTGGTCCATGCTCC |
|  | Reverse | TGCTATCACTTCCTTTCTGTTGC |
| Ccl2 | Forward | CTACTCATTCACCAGCAAGA |
|  | Reverse | TCAGCACAGACCTCTCTC |
| Il-6 | Forward | TGTATGAACAACGATGATGC |
|  | Reverse | TACTCCAGAAGACCAGAGG |

**Table S2.** Primer sequences used in cell line construction.

| Primer | Sequence | |
| --- | --- | --- |
| shAMPKα1-1 | Top | GATCCGCAGAAGTCATTTCAGGAAGATTGTATTCAAGAGATACAATCTTCCTGAAATGACTTCTGTTTTTTG |
|  | Bottom | AATTCAAAAAACAGAAGTCATTTCAGGAAGATTGTATCTCTTGAATACAATCTTCCTGAAATGACTTCTGCG |
| shAMPKα1-2 | Top | GATCCGTCTCTTTCCTGAGGACCCATCTTATTTCAAGAGAATAAGATGGGTCCTCAGGAAAGAGATTTTTTG |
|  | Bottom | AATTCAAAAAATCTCTTTCCTGAGGACCCATCTTATTCTCTTGAAATAAGATGGGTCCTCAGGAAAGAGACG |
| shAMPKα1-3 | Top | GATCCGAGCAATCAAGCAGTTGGATTATGAATTCAAGAGATTCATAATCCAACTGCTTGATTGCTTTTTTTG |
|  | Bottom | AATTCAAAAAAAGCAATCAAGCAGTTGGATTATGAATCTCTTGAATTCATAATCCAACTGCTTGATTGCTCG |
| CREB_gRNA | Forward | CACCGAGCTGCACTAAGGTTACAGT |
|  | Reverse | AAACACTGTAACCTTAGTGCAGCTC |

**Table S3.** Primer sequences used in ChIP assay.

| Region | Forward primer | Reverse primer |
| --- | --- | --- |
| ChIP | AGCCACTTACCCAGTTGAGG | TACCAGCTGGCTAGCAATACTC |
| Exon | CGAATGTTCCCCCAGCTGTT | TATTCCATTGCGCAGGCTCT |
